# Novel Three-Day, Community-Based, Nonpharmacological Group Intervention for Chronic Musculoskeletal Pain (COPERS): A Randomised Clinical Trial

**DOI:** 10.1371/journal.pmed.1002040

**Published:** 2016-06-14

**Authors:** Stephanie J. C. Taylor, Dawn Carnes, Kate Homer, Brennan C. Kahan, Natalia Hounsome, Sandra Eldridge, Anne Spencer, Tamar Pincus, Anisur Rahman, Martin Underwood

**Affiliations:** 1 Centre for Primary Care and Public Health, Queen Mary University of London, London, United Kingdom; 2 Health Economics Group, Institute of Health Research, University of Exeter, Devon, United Kingdom; 3 Department of Psychology, Royal Holloway University of London, London, United Kingdom; 4 Department of Rheumatology, University College London, London, United Kingdom; 5 Warwick Clinical Trials Unit, Warwick Medical School, Coventry, United Kingdom; Imperial College London, UNITED KINGDOM

## Abstract

**Background:**

Chronic musculoskeletal pain is the leading cause of disability worldwide. The effectiveness of pharmacological treatments for chronic pain is often limited, and there is growing concern about the adverse effects of these treatments, including opioid dependence. Nonpharmacological approaches to chronic pain may be an attractive alternative or adjunctive treatment. We describe the effectiveness of a novel, theoretically based group pain management support intervention for chronic musculoskeletal pain.

**Methods and Findings:**

We conducted a multi-centre, pragmatic, randomised, controlled effectiveness and cost-effectiveness (cost–utility) trial across 27 general practices and community musculoskeletal services in the UK. We recruited 703 adults with musculoskeletal pain of at least 3 mo duration between August 1, 2011, and July 31, 2012, and randomised participants 1.33:1 to intervention (403) or control (300). Intervention participants were offered a participative group intervention (COPERS) delivered over three alternate days with a follow-up session at 2 wk. The intervention introduced cognitive behavioural approaches and was designed to promote self-efficacy to manage chronic pain. Controls received usual care and a relaxation CD. The primary outcome was pain-related disability at 12 mo (Chronic Pain Grade [CPG] disability subscale); secondary outcomes included the CPG disability subscale at 6 mo and the following measured at 6 and 12 mo: anxiety and depression (Hospital Anxiety and Depression Scale [HADS]), pain acceptance (Chronic Pain Acceptance Questionnaire), social integration (Health Education Impact Questionnaire social integration and support subscale), pain-related self-efficacy (Pain Self-Efficacy Questionnaire), pain intensity (CPG pain intensity subscale), the census global health question (2011 census for England and Wales), health utility (EQ-5D-3L), and health care resource use. Analyses followed the intention-to-treat principle, accounted for clustering by course in the intervention arm, and used multiple imputation for missing or incomplete primary outcome data.

The mean age of participants was 59.9 y, with 81% white, 67% female, 23% employed, 85% with pain for at least 3 y, and 23% on strong opioids. Symptoms of depression and anxiety were common (baseline mean HADS scores 7.4 [standard deviation 4.1] and 9.2 [4.6], respectively). Overall, 282 (70%) intervention participants met the predefined intervention adherence criterion. Primary outcome data were obtained from 88% of participants. There was no significant difference between groups in pain-related disability at 6 or 12 mo (12 mo: difference −1.0, intervention versus control, 95% CI −4.9 to 3.0), pain intensity, or the census global health question. Anxiety, depression, pain-related self-efficacy, pain acceptance, and social integration were better in the intervention group at 6 mo; at 12 mo, these differences remained statistically significant only for depression (−0.7, 95% CI −1.2 to −0.2) and social integration (0.8, 95% CI 0.4 to 1.2). Intervention participants received more analgesics than the controls across the 12 mo. The total cost of the course per person was £145 (US$214). The cost–utility analysis showed there to be a small benefit in terms of quality-adjusted life years (QALYs) (0.0325, 95% CI −0.0074 to 0.0724), and on the cost side the intervention was a little more expensive than usual care (i.e., £188 [US$277], 95% CI −£125 [−US$184] to £501 [US$738]), resulting in an incremental cost-effectiveness ratio of £5,786 (US$8,521) per QALY. Limitations include the fact that the intervention was relatively brief and did not include any physical activity components.

**Conclusions:**

While the COPERS intervention was brief, safe, and inexpensive, with a low attrition rate, it was not effective for reducing pain-related disability over 12 mo (primary outcome). For secondary outcomes, we found sustained benefits on depression and social integration at 6 and 12 mo, but there was no effect on anxiety, pain-related self-efficacy, pain acceptance, pain intensity, or the census global health question at 12 mo. There was some evidence that the intervention may be cost-effective based on a modest difference in QALYs between groups.

**Trial registration:**

ISRCTN Registry 24426731

## Abstract 2

In a randomized controlled trial, Stephanie Taylor and colleagues investigate the effectiveness of a group intervention for chronic musculoskeletal pain.

## Author Summary

**Why Was This Study Done?:**

- Chronic pain, of which chronic musculoskeletal pain is a major component, is one of the most important causes of disability worldwide.
- Pharmacological interventions are ineffective, or only partially effective, for many people with chronic pain, and there is increasing concern about side effects (for example, with nonsteroidal anti-inflammatory drugs) or drug dependence (with opioids or gabapentinoids).
- After systematically reviewing the evidence, we developed COPERS—a novel, brief, psychologically based group intervention directed at increasing participants’ confidence and their repertoire of skills around managing pain.
- Our aim was to reduce participants’ pain-related disability.

**What Did the Researchers Do and Find?:**

- We conducted a large randomised controlled trial of the intervention, including 703 participants, mostly identified from primary care, who were randomised in a ratio of 1.33:1 to receive either the new intervention or usual care and a relaxation CD.
- The average age of the study participants was 60 y, few were in work, and most (85%) had had pain for at least 3 y; overall at baseline they reported poor health and high levels of pain-related disability, and nearly a quarter (23%) were being prescribed strong opioids.
- Compared with usual care, the intervention had no effect on pain-related disability at 6 or 12 months (the primary outcome). At 6 months, the psychological secondary outcomes were better in the group receiving the intervention compared to the group receiving usual care, but by 12 months, only depression and social integration remained improved.

**What Do These Findings Mean?:**

- This study suggests that this brief group-based psychological intervention is insufficient to improve pain-related disability in people with long-established, chronic musculoskeletal pain, and alternative treatments are needed.
- Anxiety and depression are common in people who have chronic pain, and the study holds out the tantalising prospect that the new intervention could improve the psychological well-being of such patients, but further research is needed before this conclusion can be firmly drawn.

## Introduction

Chronic pain is common, affecting an estimated 20% [1] to 30% [2] of adults worldwide. It is associated with disability, psychological comorbidity, reduced quality of life, early mortality, and high health care costs. The burden of disability due to chronic musculoskeletal disorders, commonly associated with chronic pain, increased worldwide by 46% between 1990 and 2010, with further increases predicted in coming years due to aging populations and increasing obesity [3]. In 2013 musculoskeletal disorders (combined with fractures and soft tissue injuries) accounted for over 20% of years lived with a disability across the globe [4]. Low back pain alone is the leading cause of disability in 86 countries and the second or third leading cause of disability in a further 67 countries [4].

Although pharmacological therapies have an important role in chronic pain, their effectiveness is often limited [5], and there is considerable concern about the adverse effects of nonsteroidal anti-inflammatory drugs [6,7]. Many patients with chronic pain receive opioids, despite a lack of evidence regarding their long-term effectiveness [8] and the risk of side effects, including dependence [9]. Nonpharmacological approaches to chronic pain—such as pain management and self-management support courses that aim to improve quality of life and encourage positive behaviour change—may be an attractive alternative. There are, however, limited data to support their use. There is evidence suggesting that improving self-efficacy (an individual’s belief in their ability to succeed in a particular situation) may be a key mechanism for improvement in other outcomes [10,11], placing self-efficacy as a focus of interest for self-management interventions [12].

Based on a systematic review analysing the literature on the characteristics and effectiveness of pain management programmes [13], we developed a novel, theoretically underpinned self-management support programme to improve the management of chronic musculoskeletal pain in the community, Coping with Persistent Pain, Effectiveness Research into Self-management (COPERS), and conducted a trial of this intervention [14]. The COPERS programme aimed to increase self-efficacy to manage chronic pain and attempted to address the social isolation that may accompany the experience of living with chronic pain [14]. We conceptualised the intervention within the “three-process model of pain” [15], which focuses on physiological processes, subjective-affective-cognitive processes, and behavioural processes. In this model, these are nondiscrete, interactive processes. Hence our intervention relied on changes in understanding, mood, and behaviour to enhance pain-related self-efficacy, which in turn would interact to reinforce new behaviours and affect outcomes. We hypothesised that this new intervention would reduce pain-related disability in people with chronic musculoskeletal pain. Here we describe a randomised controlled trial testing the effectiveness and cost-effectiveness of the COPERS programme.

## Methods

### Ethics Statement

The trial was overseen by independent trial steering and data monitoring and ethics committees (see Section 1 of S1 Appendix). Ethical approval was granted by the National Research Ethics Service Cambridgeshire 4 Research Ethics Committee (Ref: 11/EE/046; S1 Approval). All participants provided written informed consent before entering the trial.

### Study Participants

We conducted a pragmatic, multi-centre randomised controlled trial of the COPERS group self-management course for adults living with chronic musculoskeletal pain. Causes of pain included, but were not restricted to, osteoarthritis, back pain, chronic widespread pain, and fibromyalgia. Participants were recruited in the UK (London and the Midlands) from primary care, community musculoskeletal pain services, and secondary care pain services. The trial protocol and statistical analysis plan have been published previously [16,17].

Between August 1, 2011, and July 31, 2012, potential participants were identified via electronic patient record searches [18], face-to-face consultation, and advertisements in clinic areas. Those who responded to initial approaches or advertisement were sent a screening questionnaire. Eligibility was subsequently confirmed in a telephone interview with a researcher, who then sent potential participants a baseline questionnaire and consent form. We included adults (aged ≥18 y) with musculoskeletal pain of at least 3 mo duration [19]. Exclusion criteria were as follows: inability to give informed consent, not fluent in English, chronic pain arising from active malignant disease or inflammatory arthritis, terminal illness, or such serious, uncontrolled mental health or substance abuse issues that it would be difficult for the individual to participate in the group sessions (this was determined by the participant’s general practitioner [GP] following the electronic patient searches or through discussion between the potential participant and the researcher during the telephone interview).

### Randomisation

Following the return of completed baseline questionnaires, participants were randomised to the two groups in a 1.33:1 ratio in favour of the intervention arm. Strict allocation concealment was maintained via an independent, centralised online service that used stratified permuted blocks with randomly varying block sizes of 7 or 14 and recruitment site as a stratification factor.

### Outcome Measures

Participants completed postal questionnaires containing the outcome measures before randomisation (the baseline questionnaire) and at 6 and 12 mo following randomisation. If necessary, we collected primary outcome data by phone. Selection of outcome measures was based on their clinimetric qualities and informed by patient consultation. The primary outcome was pain-related disability at 12 mo. We chose a well-validated tool, the Chronic Pain Grade (CPG), which has two subscales—pain intensity and pain-related disability, which are scored independently and can be combined to form the CPG overall grade [20]. Each subscale has been validated separately [21]. The three disability subscale questions ask about pain interfering with daily activities; change in the ability to take part in recreational, social, and family activities; and change in the ability to work (including housework) over the past 6 mo [20,21]. To generate the outcome, each item is scored on a scale 0–10 (with 10 the worst), and the mean is taken and multiplied by 100. The CPG disability subscale has been used in a number of other trials investigating long-term pain [22,23]. Secondary outcomes were the CPG disability subscale at 6 mo and the following at 6 and 12 mo: pain intensity (CPG pain intensity subscale) [20,21], the census global health question (2011 census for England and Wales) [24], anxiety and depression (Hospital Anxiety and Depression Scale [HADS]) [25], pain acceptance (Chronic Pain Acceptance Questionnaire [CPAQ]) [26], social integration (Health Education Impact Questionnaire [heiQ] social integration and support subscale) [27], health utility (EQ-5D-3L) [28], pain-related self-efficacy (Pain Self-Efficacy Questionnaire [PSEQ]) [29], and health care resource use. We also examined use of psychotropic medicines, analgesics, and weak and strong opioids by looking at total World Health Organization defined daily doses (DDDs) of selected medications prescribed in the 12 mo following randomisation and the proportion of participants using strong and weak opioids at 12-mo follow-up. Full details of our outcome measures and methods are described in Sections 2–4 of S1 Appendix. Due to the nature of the intervention, it was not feasible to mask participants or group facilitators to study arm. Participants’ health care professionals and all those retrieving, handling, or processing outcome data remained unaware of participants’ allocated study arms.

Adverse events for both arms were collected following standard operating procedures for the Pragmatic Clinical Trials Unit and our adverse events protocol. Adverse events in the control arm could be reported by participants at any time via phone or post, and we also reviewed all medical records at the end of the study. All deaths occurring during the study period were scrutinised to determine if they were related to the study.

### Intervention

The intervention was a facilitated, experiential learning group course based on cognitive behavioural principles plus usual care (Table 1); its development and content is described in detail elsewhere [14]. Briefly, the course consisted of 24 individual components delivered in a community setting over three alternate days in 1 wk, with a follow-up session 2 wk later (total duration = 14 h). Content included cognitive behavioural approaches to managing chronic pain (these covered acceptance, attention control, goal setting and action planning, and recognising unhelpful thinking and behaviours); an educational DVD with a pain consultant answering common questions from a patient with chronic pain; communication skills; relationships; hobbies and activities; posture and movement; and breathing, relaxation, and guided imagery. Courses were delivered by two facilitators: a health care professional with experience treating people with chronic musculoskeletal pain (physiotherapist, psychologist, osteopath, or GP) and a lay person living with chronic pain. Following a 2-d joint training programme, facilitators who met predetermined competence criteria were selected to deliver the intervention. All courses were audio recorded, and a random selection of the recordings of particular components from each course was analysed to evaluate intervention fidelity, described in detail elsewhere [30]. Participants present for at least 17 of the 24 course components were deemed “adherent” to the intervention, according to a predetermined criterion.

**Table 1:** Outline of the intervention—the COPERS course.

Day: Topic	Modules	Content of Sessions
**1: Living and dealing with pain**	1. Introduction and understanding pain and acceptance	•Component 1: Introduction
		•Component 2: Pain information
		•Component 3: Acceptance—the uninvited guest
	Lunch	
	Taster activity—art	
	2. Mind, mood, and pain	•Component 4: Pain—when is it bearable and when is it not?
		•Component 5: The pain cycle
	3. Movement and relaxation	•Component 6: Posture
		•Component 7: Relaxation and breathing
**2: Doing something about your life with pain**	4. Dealing with unhelpful negative thoughts and barriers to change	•Component 8: Reflections from day one
		•Component 9: Identifying problems, goal setting, and action planning
		•Component 10: Barriers to change—unhelpful thinking
	Lunch	
	Taster activity—hand massage	
	5. Making pain more manageable	•Component 11: Barriers to change—reframing negatives to positives
		•Component 12: Attention control and distraction
		•Component 13: Things that make pain more manageable
	6. Movement and relaxation	•Component 14: Balance and stretch
		•Component 15: Relaxation and visualisation
**3: Communication and relationships**	7. Communication skills	•Component 16: Reflections from day 2
		•Component 17: Communicating with your GP
		•Component 18: Listening skillsComponent 19: Anger, irritability, and frustration
	Lunch	
	Taster activity—volunteering	
	8. Movement and relaxation	•Component 20: Stretch
		•Component 21: Relaxation and mindfulness of thoughts
		•Component 22. Summary of the course
**4: Follow-up**	9. The future	•Component 23: Reflections and feedback from the group
		•Component 24: Managing setbacks

### Usual Care

The control group received usual care (including a widely available pain education leaflet: http://www.paintoolkit.org/downloads/SC_TK_NHS_TAYSIDE.pdf) and a relaxation CD (also given to intervention participants). To mimic the duration of the intervention, control participants were asked to practise relaxation daily for 3 wk and whenever they wished thereafter.

### Statistical Analyses

To show a standardised mean difference (SMD) (mean difference divided by the standard deviation at baseline) in pain-related disability of 0.3 between the intervention and control groups, at a 5% significance level with 80% power, would require data from 350 participants. To minimise the overall sample size in a situation where clustering occurred only in the intervention arm (due to the group intervention), we used Moerbeek’s method, inflating the sample size by 1.37 (assuming an intracluster correlation coefficient of 0.1 and nine participants per course providing 12-mo follow-up data) and using an unbalanced randomisation (1.33:1 in favour of the intervention) [31]. Using this approach, we required data from 480 individuals. Allowing for a 30% loss to follow-up, we sought to randomise 685 participants (391:294).

All analyses were performed according to the intention-to-treat principle. All participants with an available outcome were analysed according to the group to which they were randomised. All analyses accounted for clustering by course in the intervention arm through use of a random effect in a mixed-effects regression model (with participants in the control arm acting as their own cluster) [32]. Treatment group, age, gender, site of recruitment (London or Midlands) [33–35], and baseline level of outcome were included in each analysis as fixed effects [36].

We used multiple imputation for analysis of the primary outcome, pain-related disability [34]. We imputed the individual questions that formed the CPG disability score and therefore included in the imputation model and in the analysis all participants who answered at least one question on the CPG disability subscale at either 6 or 12 mo. Participants who did not answer any questions on the CPG subscale at either 6 or 12 mo were excluded from the analysis. We used multilevel imputation, with course included in the imputation model as a random effect. The imputation model included the three questions that formed the CPG disability score at baseline, 6 mo, and 12 mo, as well as site of recruitment, age, gender, HADS depression score at baseline, and employment status. Imputation was conducted separately within each treatment group, and 20 imputations were performed (i.e., we created 20 complete datasets). We analysed outcomes at 6 and 12 mo separately, using a mixed-effects linear regression model as described above. Results were combined using Rubin’s rules [37]. Analysis of secondary outcomes is described in Section 4 of S1 Appendix.

We performed sensitivity analyses to assess the robustness of the analysis to different assumptions regarding missing data (methods described in Section 5 of S1 Appendix). We performed preplanned subgroup analyses for the primary outcome (full details in Section 11 of S1 Appendix) based on the following characteristics: number of comorbidities, living arrangement (living alone or not), baseline PSEQ score, socioeconomic status, pain duration, baseline CPG pain intensity score, baseline CPG disability score, and baseline HADS depression score. Subgroup analyses were performed by including an interaction between the specified characteristic and the treatment arm in the analysis. Full details of the statistical methods can be found in the analysis plan (including details for all subgroup analyses, sensitivity analyses, and analyses of secondary outcomes) [17], which was finalised before any investigators had unmasked access to trial data. All analyses presented here were predefined in the statistical analysis plan [17] unless otherwise stated. A list of deviations from the analysis plan is available in Section 6 of S1 Appendix. Analysis was performed using Stata version 13 and REALCOM-IMPUTE software [38].

### Health Economic Analysis

The health economic analysis took a health care provider perspective and estimated the costs of delivering the intervention and all further primary, secondary, and community care costs (see Section 7 of S1 Appendix for more details on methods). Service use data, including all prescribing data, were collected from participants’ GP electronic records at 12-mo follow-up. Data relating to secondary care use was downloaded from the Secondary Uses Service database [39]. Resource use data were combined with unit costs to calculate the total cost of health service use for each participant (see Section 8 of S1 Appendix for unit costs). Missing data for costs and quality-adjusted live years (QALYs) were imputed using Stata 12.1. The primary economic analysis was a cost–utility analysis over 12 mo using QALYs calculated from the EQ-5D-3L. We used a mixed-effects linear regression model to adjust estimates of costs and QALYs for baseline measures, treatment group, age, gender, and site of recruitment as fixed effects and course as a random effect (with participants in the control arm acting as their own cluster). We used nonparametric bootstrapping and multiple imputations to compute cost-effectiveness acceptability curves and assessed cost–utility using willingness to pay thresholds ranging between £0 and £30,000 (US$44,183). Costs were converted to US dollars using the purchasing power parity rate (2013) (OECD.Stat; http://stats.oecd.org/).

## Results

### Study Participants

**Fig 1:**
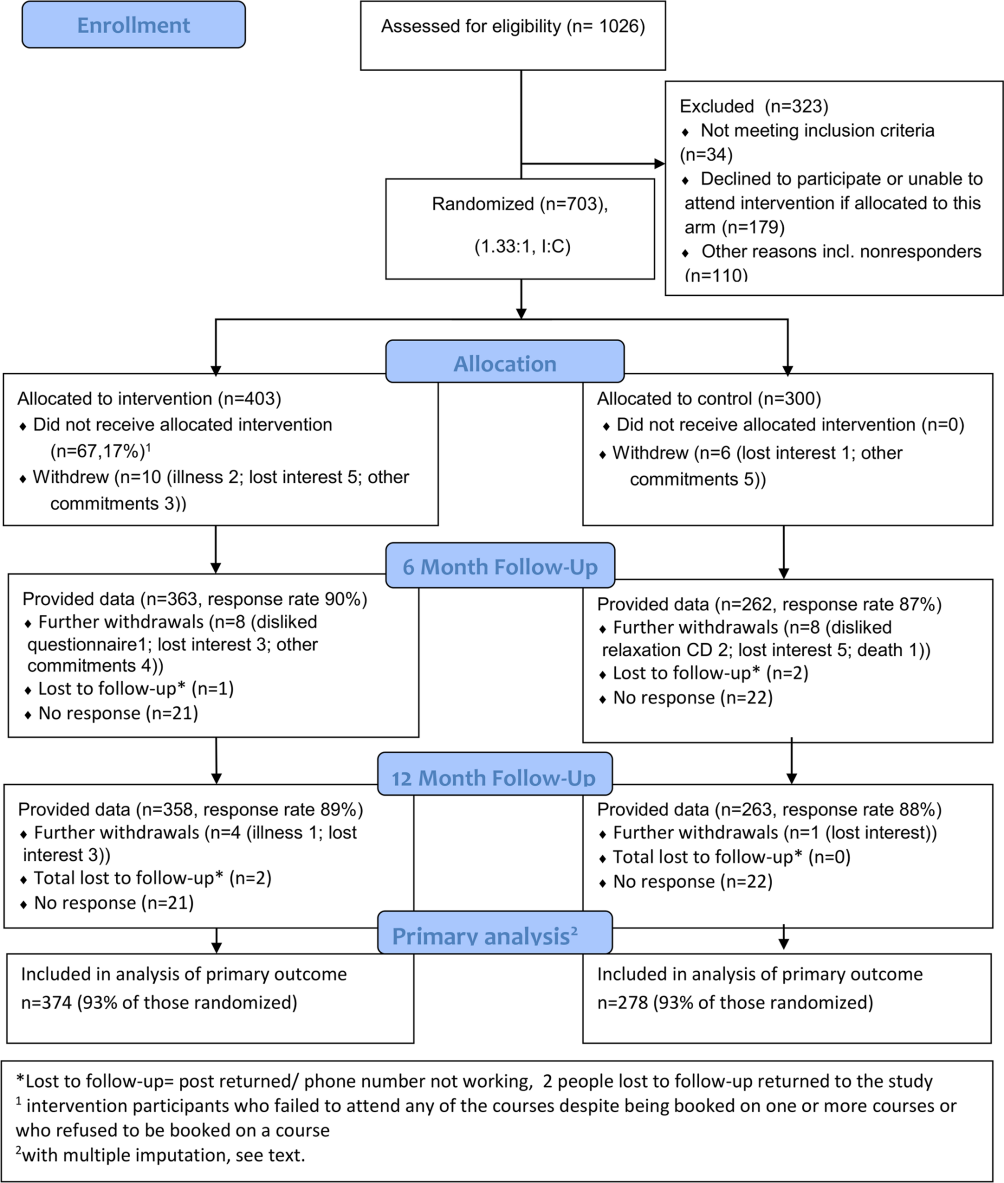
COPERS CONSORT flow chart. I:C, intervention: control.

Between August 1, 2011, and July 31, 2012, we randomised 703 participants from 35 general practices, two secondary care pain services, and one community-based musculoskeletal service (403:300, intervention:control) (Fig 1).

We over-recruited to ensure that the final self-management support groups at all study centres achieved the prespecified minimum number of attending participants (five). Intervention and control participants were well matched at baseline (Table 2). Most of the participants (85%) had had pain for at least 3 y, with 265 (38%) reporting pain for more than 10 y and 162 (23%) being prescribed strong opioids (as defined in the British National Formulary [40]) at baseline. The median number of comorbidities (determined from primary care records) was 2 (range 0–8). Only 169 (24%) participants were in any form of employment, with 148 (21%) who were unable to work due to long-term sickness and another 307 (44%) who were retired. Overall health utility as assessed by the EQ-5D-3L (commonly interpreted as quality of life) was very low (mean 0.4, standard deviation [SD] 0.34).

**Table 2:** Baseline characteristics.

Characteristic	Control (*n* = 300)	Intervention (*n* = 403)	Number of Participants with Missing Data (Control, Intervention)
**Age (years)**	59.4 (13.8)	60.3 (13.5)	0, 0
**Male**	98 (33%)	132 (33%)	0, 0
**Lives alone**	101 (34%)	143 (36%)	4, 6
**Ethnicity**			0, 0
White	239 (80%)	325 (81%)	
Black	36 (12%)	53 (13%)	
Asian	20 (7%)	13 (3%)	
Mixed/other	5 (<1%)	12 (3%)	
**Age at which formal education ended**			0, 0
16 y old or younger	157 (52%)	224 (56%)	
20 y old or older	135 (45%)	173 (43%)	
Other	8 (3%)	6 (1%)	
**Employment status**			0, 0
Employed, including self-employed (full or part time)^a^	95 (32%)	115 (29%)	
Unemployed looking for work or unable due to long-term sickness	72 (24%)	106 (26%)	
Retired from paid work	132 (44%)	175 (43%)	
Other	1 (<1%)	7 (2%)	
**Time kept from usual activities due to pain in past 6 mo**			3, 3
0–6 d	84 (28%)	136 (34%)	
7–14 d	49 (17%)	72 (18%)	
15–30 d	57 (19%)	71 (18%)	
31 d or more	107 (36%)	121 (30%)	
**State of health** ^**b**^			0, 0
Very good	17 (6%)	27 (7%)	
Good	100 (33%)	138 (34%)	
Fair	130 (43%)	159 (39%)	
Bad	45 (15%)	63 (16%)	
Very bad	8 (3%)	16 (4%)	
**Duration of pain**			0, 0
0–3 mo	4 (1%)	1 (<1%)	
4–12 mo	10 (3%)	15 (4%)	
13 mo—2 y	43 (14%)	45 (11%)	
3–4 y	45 (15%)	55 (14%)	
5–6 y	40 (13%)	49 (12%)	
7–10 y	50 (17%)	81 (20%)	
More than 10 y	108 (36%)	157 (39%)	
**CPG [20] overall grade** ^**c**^			3, 5
0	0 (0%)	0 (0%)	
1	18 (6%)	30 (8%)	
2	66 (22%)	99 (25%)	
3	81 (27%)	123 (31%)	
4	132 (44%)	146 (37%)	
**CPG [20] disability** ^**d**^	63.8 (24.4)	62.9 (25.7)	0, 1
**CPG [20] pain intensity** ^**e**^	70.9 (15.3)	71.5 (17.0)	1, 1
**PSEQ [29]** ^**f**^	30.6 (14.1)	31.2 (13.8)	0, 5
**CPAQ [26]** ^**g**^	55.3 (19.1)	57.5 (20.7)	7, 15
**HADS depression [25]** ^**h**^	7.5 (4.0)	7.4 (4.2)	3, 2
**HADS anxiety [25]** ^**i**^	9.3 (4.7)	9.2 (4.6)	3, 3
**HADS [25] depression score categories**			3, 2
0–7 (normal)	159 (54%)	217 (54%)	
8–10 (mild)	74 (25%)	95 (24%)	
11–21 (moderate or severe)	64 (22%)	89 (22%)	
**heiQ [27] social integration and support** ^**j**^	13.8 (3.4)	14.0 (3.6)	5, 3
**EQ-5D-3L [28]** ^**k**^	0.39 (0.34)	0.41 (0.34)	1, 1
**Number of comorbidities**	3 (2 to 4)	2 (2 to 3)	21, 32

Data are number (percent) or mean (SD) unless otherwise indicated.

^a^Includes in full-time education and looking after home/family.

^b^UK census general health question [24].

^c^CPG overall grades 0 (no pain) to 4 (high disability, severely limiting pain).

^d^CPG pain disability subscale: mean CPG disability items scored on a scale 0–10 (worst) and multiplied by 100, thus 100 is the worst possible score.

^e^CPG pain intensity subscale: mean of the three pain intensity CPG items scored on a scale 0–10 (worst) and multiplied by 100.

^f^PSEQ score: 0–60 (best).

^g^CPAQ score: 0–120 (best).

^h^HADS depression score: 0–21 (worst).

^i^HADS anxiety score: 0–21 (worst).

^j^heiQ social integration and support subscale: 4–20 (best).

^k^EQ-5D-3L: <0–1 (best).

^l^Median (interquartile range); from primary care records.

Eleven health care professionals and 13 lay people delivered 35 courses (the mean number of participants per course was 14). The mean waiting time from randomisation to attending a course was 6 wk (range 0–24 wk); 67/403 (17%) intervention participants did not attend a course, and 282 (70%) met our predefined definition of adherence.

### Pain-Related Disability

We obtained a complete set of baseline and primary outcome data from 621 (88%) participants and, with multiple imputation for missing primary outcome data (see above), were able to include 652 (93%) participants in our analysis (Fig 1). Table 3 shows the results for primary and secondary outcomes at 6- and 12-mo follow-up. Pain-related disability did not differ between treatment groups at either time (12 mo: intervention mean 52.9 [SD 28.0] versus control mean 53.3 [SD 28.8]; difference [intervention minus control] −1.0, 95% CI −4.9 to 3.0).

**Table 3:** Main results for primary and secondary outcomes.

Outcome	Mean (SD)^a^		Treatment Effect^b^ (95% CI)	
	Control (*n* = 300)	Intervention (*n* = 403)	Difference in Means (Intervention Minus Control)	SMD^c^
**CPG [20] disability subscale**				
6 mo	54.3 (26.7)	53.2 (25.7)	−1.2 (−4.8 to 2.4)	−0.06 (−0.24 to 0.12)
12 mo	53.3 (28.8)	52.9 (28.0)	−1.0 (−4.9 to 3.0)	−0.04 (−0.22 to 0.13)
**CPG [20] pain intensity subscale**				
6 mo	64.3 (19.4)	65.0 (18.8)	1.0 (−1.5 to 3.6)	0.07 (−0.10 to 0.24)
12 mo	64.4 (20.1)	63.5 (20.3)	−0.9 (−3.7 to 1.9)	−0.06 (−0.23 to 0.12)
**PSEQ [29] score**				
6 mo	32.7 (15.0)	35.5 (14.0)	2.3 (0.6 to 4.1)	0.25 (0.07 to 0.43)
12 mo	33.4 (15.1)	35.4 (14.1)	1.4 (−0.2 to 3.1)	0.15 (−0.02 to 0.32)
**HADS [25] anxiety score**				
6 mo	9.1 (4.8)	8.2 (4.7)	−0.7 (−1.3 to −0.2)	−0.24 (−0.41 to −0.06)
12 mo	8.4 (4.5)	8.1 (4.5)	−0.4 (−0.9 to 0.1)	−0.13 (−0.30 to 0.03)
**HADS [25] depression score**				
6 mo	7.0 (4.4)	6.3 (4.1)	−0.7 (−1.2 to −0.2)	−0.25 (−0.44 to −0.06)
12 mo	6.9 (4.6)	6.2 (4.3)	−0.7 (−1.2 to −0.2)	−0.22 (−0.39 to −0.06)
**CPAQ [26] score**				
6 mo	59.2 (19.7)	64.4 (20.0)	3.4 (1.3 to 5.5)	0.27 (0.08 to 0.45)
12 mo	74.0 (14.4)	73.1 (15.1)	−0.8 (−3.0 to 1.4)	−0.03 (−0.20 to 0.13)
**heiQ [27] social integration and support subscale**				
6 mo	14.3 (3.6)	14.9 (3.3)	0.6 (0.1 to 1.0)	0.25 (0.06 to 0.43)
12 mo	14.1 (3.6)	14.9 (3.5)	0.8 (0.4 to 1.2)	0.32 (0.16 to 0.49)
**EQ-5D-3L [28]**				
6 mo	0.41 (0.35)	0.46 (0.34)	0.03 (−0.01 to 0.08)	0.13 (−0.03 to 0.29)
12 mo	0.45 (0.35)	0.46 (0.34)	0.00 (−0.04 to 0.04)	0.01 (−0.16 to 0.17)

^a^Means (SD) for both treatment groups are based on raw data, i.e., are unadjusted.

^b^The difference in means and the SMD were adjusted for age, gender, site of recruitment (London or Midlands), and baseline level of outcome.

^c^SMDs were calculated using the residual SD obtained from the analysis model.

### Secondary Outcomes

At 6 mo, pain-related self-efficacy (PSEQ, difference 2.3, 95% CI 0.6 to 4.1), anxiety (HADS anxiety subscale, −0.7, 95% CI −1.3 to −0.2), depression (HADS depression subscale, −0.7, 95% CI −1.2 to −0.2), pain acceptance (CPAQ, 3.4, 95% CI 1.3 to 5.5), and social integration (heiQ social integration and support subscale, 0.6, 95% CI 0.1 to 1.0) had all improved more in the intervention group than in the control group (Table 3). At 12 mo, the differences favouring the intervention were sustained for depression (−0.7, 95% CI −1.2 to −0.2) and social integration (0.8, 95% CI 0.4 to 1.2). All sensitivity analyses had results similar to those of the primary analysis, demonstrating that primary outcome results were robust (see Section 9 of S1 Appendix for full results).

There was no difference between the intervention and control groups in response to the census global health question at 6- or 12-mo follow-up (odds ratio for intervention group participants reporting improved state of health at 12 mo was 1.07, 95% CI 0.77 to 1.51). Overall, intervention patients received considerably more analgesics than controls in the 12 mo following randomisation (amounting to an average difference of 98 d [95% CI 17 to 178] of medication at WHO DDD). They also received significantly more weak opioids (18 d, 95% CI 5 to 32, at DDD). However, there was no evidence of any difference in the prescription of strong opioids between treatment arms (−1 d, 95% CI −12 to 11, at DDD) nor in the proportions of those receiving strong opioids at 12 mo (see Section 10 of S1 Appendix for full results).

No serious adverse events occurred in the intervention or control arms. Two deaths occurred during the study: one intervention patient and one control patient. Both deaths were considered by the chair of our data monitoring and ethics committee to be unrelated to the study and thus not to represent adverse events.

Prespecified subgroup analyses—examining subgroups based on number of comorbidities, living arrangement, baseline PSEQ score, socioeconomic status, pain duration, baseline CPG pain intensity score, baseline CPG disability score, and baseline HADS depression score—found no differences across subgroups (full results in Section 11 of S1 Appendix) for the primary outcome. An exploratory post hoc subgroup analysis found that improvement in 12-mo depression score occurred only in those who were likely to be depressed at baseline (*p-*value for interaction 0.004) (Table 4).

**Table 4:** Subgroup analysis of HADS depression score at 12 mo by HADS depression score at baseline: 0–7 versus 8–21.

HADS Depression Score at Baseline	Mean (SD)		Treatment Effect (95% CI)	*p*-Value for Interaction
	Control	Intervention		
**Original scale**				0.004
0–7	4.2 (3.0)	4.0 (3.0)	0.0 (−0.7 to 0.6)	
8–21	9.4 (4.8)	8.2 (4.7)	−1.5 (−2.3 to −0.8)	
**SMD**				—
0–7	—	—	−0.01 (−0.23 to 0.21)	
8–21	—	—	−0.50 (−0.74 to −0.25)	

A total of 625 participants were included in the subgroup analysis: 348 patients with HADS depression score 0–7 (148 control, 200 intervention) and 277 patients with HADS depression score 8–21 (113 control, 164 intervention).

### Health Economic Analyses

We obtained complete health economics data from 540 participants (77% of participants). The highest proportion of missing data was for baseline prescriptions, followed by EQ-5D-3L and primary care contacts. Imputing the data for missing values resulted in a dataset of 647 participants (92%) (control *n* = 275, intervention *n* = 372), which represented 99% of the trial population included in the statistical analyses of the primary outcome. The cost of delivering courses, including the cost of training the facilitators, was £145 (US$214) per person. Total costs were higher in the intervention group (£2,955, US$4,352) compared to the control group (£2,767, US$4,075), and the difference in the means was £188 (US$277), 95% CI −£125 (−US$184) to £501 (US$738). Total QALYs were also higher in the intervention group (0.4475) compared to the control group (0.4150), and the difference in the means was 0.0325 (95% CI −0.0074; 0.0724) QALYs. The incremental cost-effectiveness ratio mean point estimate was £5,786 (US$8,521) per QALY. The intervention had a high probability (87%) of being cost-effective at a willingness to pay of £30,000 (US$44,183) per QALY. Detailed results of cost-effectiveness analyses are shown in Section 12 of S1 Appendix.

## Discussion

Our chronic pain self-management intervention (COPERS) was relatively cheap to deliver and had a good uptake (336/403, 86%), with little attrition. We found no evidence of an impact of the intervention on our primary outcome of pain-related disability at 12 mo, or in pain-related disability at 6 mo. However, at 6 mo, the COPERS intervention led to improved psychological well-being compared to the control group with regard to social integration and all our psychological measures—anxiety, depression, pain acceptance, and pain-related self-efficacy. At 12 mo, the intervention arm showed continued beneficial effects for depression and social integration. These changes in health-related quality of life were reflected in an incremental gain in QALYs of 0.035—a gain that is similar in size to that observed in other patient self-management programmes [41,42]—and the intervention did not result in any adverse events. The intervention was also relatively low cost, resulting in a mean cost of £5,786 (US$8,521) per QALY. There is uncertainty around the estimates of costs and QALYs, but when we took account of this uncertainty, the intervention was shown to have a high probability (87%) of being cost-effective at the current UK National Institute for Health and Care Excellence threshold of £30,000 (US$44,183) per QALY [43].

The finding of a long-term effect on the secondary outcome of depression is of some interest. Nearly half our participants, 322/703 (46%), met the criterion for possible clinical depression at baseline [44]. Our observed overall effect size for depressive symptoms exceeds the effect size found in an individual patient data meta-analysis of selective serotonin reuptake inhibitors for mild/moderate depression (SMD 0.11, 95% CI −0.18 to 0.41) or severe depression (SMD 0.17, 95% CI −0.08 to 0.43) [45].

An exploratory post hoc analysis found a clinically significant, sustained improvement in depressive symptoms at 12 mo amongst participants with depressive symptoms at baseline, with no benefit for those who did not meet this depression criterion at baseline. In this post hoc analysis, the SMD gain from our intervention in the subgroup meeting the depression criterion at baseline (−0.50, 95% CI −0.74 to −0.25) was of a similar size to SMD gains reported in a network meta-analysis of large trials (≥50 per group) of psychotherapeutic interventions for depression [46]: interpersonal therapy (−0.73, 95% CI −1.14 to 0.32), cognitive behavioural therapy (−0.47, 95% CI −0.80 to −0.35), and problem-solving therapy (−0.46, 95% CI −0.81 to −0.12), and to in the SMD gain reported in a Cochrane review of tricyclic antidepressants in primary care (−0.49, 95% CI −0.67 to −0.32) [47]. Notwithstanding these promising results, the COPERS intervention cannot be recommended for people with depressive symptoms associated with musculoskeletal pain without evidence that this effect is found in a study including only those with depressive symptoms.

The key strengths of this study were its pragmatic design, the lack of attrition, and the robustness of the results. We used multiple imputation to include all participants with follow-up data in the analysis, and conducted extensive sensitivity analyses to confirm the robustness of our results. Before we analysed the trial outcome, we evaluated the fidelity of our intervention—this showed that it was delivered as intended [30].

All outcomes in both the intervention and control groups improved over time (Tables 2 and 3). The inclusion of a relaxation CD and leaflet along with usual care in the control arm might have reduced the apparent effectiveness of the intervention. We chose the relaxation package because other studies had suggested that although relaxation was popular, it was unlikely to have an effect on our primary outcome of pain-related disability or have long-term effects, but we cannot exclude the possibility that it had a therapeutic effect [15].

It's not clear why participants in the intervention group were prescribed more pain medications than those in the control group. This finding might have arisen as a result of the former gaining greater confidence or skill in communicating with their health professionals (an explicit aim of the intervention). The COPERS intervention could be more effective if it was combined with an intervention that attempted to optimise analgesic prescribing for each individual (a strategy we are currently investigating in chronic headache in the Chronic Headache Education and Self-management Study; http://www2.warwick.ac.uk/fac/med/research/hscience/ctu/trials/other/chess/).

A review and meta-analysis of mediation studies of people with back and neck pain found evidence that self-efficacy, psychological distress, and fear (principally fear of movement) may explain the development of disability in people with low back or neck pain [48], although the studies were noted to be of low quality. In our study, improvements in self-efficacy and psychological distress were *not* accompanied by a reduction in self-reported pain-related disability. It is possible that our intervention was too brief to have an effect on pain outcomes in this population, which, overall, reported a long history of pain, high levels of pain-related disability, and low quality of life at baseline, but we were able to demonstrate a sustained effect on psychological outcomes. Most psychological interventions recognise that while improvements in pain in patients with longstanding pain are unlikely, improving function and well-being are paramount. Our intervention performed as well as cognitive behavioural therapy for chronic pain [49].

Improving pain-related disability may require more intensive exercise-based interventions, whilst this intervention was devised to encourage behaviour change for long-term lifestyle change. Using this type of intervention as an adjunctive treatment may be optimal, for example, with a stepped care analgesic algorithm, such as in the SCOPE trial [50].

Although this brief intervention appears to be inexpensive and safe, and had a good uptake and low attrition, it did not improve the primary outcome of pain-related disability. The intervention’s potential to improve the psychological well-being of people with chronic pain, many of whom may also be anxious or depressed, could benefit large numbers of people with chronic pain, but requires further research. Currently, it is difficult to justify the intervention’s use for those without depression, and we do not know its effectiveness if only people with probable depression are included in the group intervention.

### Conclusion

This novel, theoretically based intervention did not improve pain-related disability in people with chronic musculoskeletal pain. It may have a valuable role in promoting psychological well-being amongst people with chronic pain who are also anxious or depressed, but this needs further research. Moreover, effective interventions to improve hard to shift outcomes, such as disability, in chronic pain patients are still required.

## Supporting Information

S1 CONSORT Checklist CONSORT 2010 checklist of information to include when reporting a randomised trial. (DOCX)

S1 Appendix Supplementary appendices. (PDF)

S1 Approval Final approval letter from National Research Ethics Service Cambridgeshire 4 Research Ethics Committee. (PDF)

S1 Protocol Coping with Persistent Pain, Effectiveness Research for Self-management: a randomised controlled trial (protocol). (PDF)

## Abbreviations

CPAQ: Chronic Pain Acceptance Questionnaire
CPG: Chronic Pain Grade
DDD: defined daily dose
GP: general practitioner
HADS: Hospital Anxiety and Depression Scale
heiQ: Health Education Impact Questionnaire
PSEQ: Pain Self-Efficacy Questionnaire
QALY: quality-adjusted life year
SD: standard deviation
SMD: standardised mean difference

## References

[1] Smith BH, Torrance N. Epidemiology of chronic pain In: McQuay HJ, Kalso E, Moore RA, editors. Systematic reviews in pain research: methodology refined. Seattle: IASP Press; 2008 pp. 247–274.

[2] Elzahaf RA, Tashani OA, Unsworth BA, Johnson MI. The prevalence of chronic pain with an analysis of countries with a Human Development Index less than 0.9: a systematic review without meta-analysis. Curr Med Res Opin. 2012;28:1221–1229. 10.1185/03007995.2012.703132 22697274

[3] Murray CJ, Vos T, Lozano R, Naghavi M, Flaxman AD, Michaud C, et al Disability-adjusted life years (DALYs) for 291 diseases and injuries in 21 regions, 1990–2010: a systematic analysis for the Global Burden of Disease Study 2010. Lancet. 2012;380:2197–2223. 10.1016/S0140-6736(12)61689-4 23245608

[4] Global Burden of Disease Study 2013 Collaborators. Global, regional, and national incidence, prevalence, and years lived with disability for 301 acute and chronic diseases and injuries in 188 countries, 1990–2013: a systematic analysis for the Global Burden of Disease Study 2013. Lancet. 2015;386:743–800. 10.1016/S0140-6736(15)60692-4 26063472 PMC4561509

[5] Moore A, Derry S, Eccleston C, Kalso E. Expect analgesic failure; pursue analgesic success. BMJ. 2013;346:f2690 10.1136/bmj.f2690 23645858

[6] McAlindon TE, Bannuru RR, Sullivan MC, Arden NK, Berenbaum F, Bierma-Zeinstra SM, et al OARSI guidelines for the non-surgical management of knee osteoarthritis. Osteoarthritis Cartilage. 2014;22:363–388. 10.1016/j.joca.2014.01.003 24462672

[7] Lanas A, Benito P, Alonso J, Hernández-Cruz B, Barón-Esquivias G, Perez-Aísa Á, et al Safe prescription recommendations for non steroidal anti-inflammatory drugs: consensus document elaborated by nominated experts of three scientific associations (SER-SEC-AEG). Reumatol Clin. 2014;10:68–84. 24462644 10.1016/j.reuma.2013.10.004

[8] Chaparro LE, Furlan AD, Deshpande A, Mailis-Gagnon A, Atlas S, Turk DC. Opioids compared to placebo or other treatments for chronic low-back pain. Cochrane Database Syst Rev. 2013;8:CD004959 10.1002/14651858.CD004959.pub4 23983011 PMC11056234

[9] Olsen Y, Sharfstein J. Chronic pain, addiction, and Zohydro. N Engl J Med. 2014;370:2061–2063. 10.1056/NEJMp1404181 24758596

[10] Miles CL, Pincus T, Carnes D, Homer KE, Taylor SJC, Bremner SA, et al Can we identify how programmes aimed at promoting self-management in musculoskeletal pain work and who benefits? A systematic review of sub-group analysis within RCTs. Eur J Pain. 2011;15:1–11. 21354838 10.1016/j.ejpain.2011.01.016

[11] Ashford S, Edmunds J, French DP. What is the best way to change self-efficacy to promote lifestyle and recreational physical activity? A systematic review with meta-analysis. Br J Health Psychol. 2010;15:265–288. 10.1348/135910709X461752 19586583

[12] Lorig KR, Holman H. Self-management education: history, definition, outcomes, and mechanisms.Ann Behav Med. 2003;26:1–7. 12867348 10.1207/S15324796ABM2601_01

[13] Carnes D, Homer KE, Miles CL, Pincus T, Underwood M, Rahman A, et al Effective delivery styles and content for self-management interventions for chronic musculoskeletal pain: a systematic literature review. Clin J Pain. 2012;28:344–354. 10.1097/AJP.0b013e31822ed2f3 22001667

[14] Carnes D, Homer K, Underwood M, Pincus T, Rahman A, Taylor SJC. Pain management for chronic musculoskeletal conditions: the development of an evidence based and theory informed pain self-management course. BMJ Open. 2013;3:e003534 10.1136/bmjopen-2013-003534 24231458 PMC3831098

[15] Ogden J. Health psychology: a textbook Maidenhead (United Kingdom): Open University Press; 2012.

[16] Carnes D, Underwood M, Homer K, Eldridge S, Bremner S, Pincus T, et al Effectiveness and cost effectiveness of a novel, group self-management course for adults with chronic musculoskeletal pain: study protocol for a multicentre, randomised controlled trial (COPERS). BMJ Open. 2013;3:e002492 10.1136/bmjopen-2012-002492 23358564 PMC3563130

[17] Kahan B, Diaz-Ordaz K, Homer K, Carnes D, Underwood M, Taylor SJ, et al Coping with persistent pain, effectiveness research into self-management (COPERS)—statistical analysis plan for a randomised controlled trial. BMC Trials. 2014;15:59 10.1186/1745-6215-15-59 PMC3930300 24528484

[18] Foell J, Carnes D, Homer K, Taylor SJC. Developing and implementing electronic search strategies to recruit patients with chronic musculoskeletal pain in primary care databases. Prim Health Care Res Dev. 2014;15:234–243. 10.1017/S1463423613000248 23702299

[19] Mersky H, Bogbuk N, editors. Classification of chronic pain descriptions of chronic pain syndromes and definitions of pain terms 2nd edition Seattle: IASP Press; 1994.

[20] Von Korff M, Ormel J, Keefe FJ, Dworkin SF. Grading the severity of chronic pain. Pain. 1992;50:133–149. 1408309 10.1016/0304-3959(92)90154-4

[21] Smith BH, Penny KI, Purves AM, Munro C, Wilson B, Grimshaw J, et al The Chronic Pain Grade questionnaire: validation and reliability in postal research. Pain. 1997;71:141–147. 9211475 10.1016/s0304-3959(97)03347-2

[22] Lamb SE, Lall R, Hansen Z, Castelnuovo, Withers EJ, Nichols V, et al A multicentred randomised controlled trial of a primary care-based cognitive behavioural programme for low back pain. The Back Skills Training (BeST) trial. Health Technol Assess. 2010;14:1–253. 10.3310/hta14410 20807469

[23] UK BEAM trial team. UK back pain, exercise and manipulation (UK BEAM) randomised trial: effectiveness of physical therapies for back pain in primary care. BMJ 2004;329:1377–1383. 15556955 10.1136/bmj.38282.669225.AE PMC535454

[24] Office for National Statistics. 2011 census. [cited 9 May 2016]. Available: https://www.ons.gov.uk/census/2011census.

[25] Zigmond AS, Snaith RP. The hospital anxiety and depression scale. Acta Psychiatr Scand. 1983;67:361–370. 6880820 10.1111/j.1600-0447.1983.tb09716.x

[26] McCracken LM, Vowles KE, Eccleston C. Acceptance of chronic pain: component analysis and a revised assessment method. Pain. 2004;107:159–166. 14715402 10.1016/j.pain.2003.10.012

[27] Deakin University. Measurement to generate change: Health Education Impact Questionnaire (heiQ). 2015 Nov 9 [cited 9 May 2016]. Available: http://www.deakin.edu.au/research/cphr/our-research/public-health-innovation-unit/our-research.

[28] EuroQol Group. About EQ-5D. 2016 [cited 9 May 2016]. Available: http://www.euroqol.org/about-eq-5d.html.

[29] Nicholas MK. The pain self-efficacy questionnaire: taking pain into account. Eur J Pain. 2007;11:153–163. 16446108 10.1016/j.ejpain.2005.12.008

[30] Mars T, Ellard D, Carnes D, Homer K, Underwood M, Taylor SJ. Fidelity in complex behaviour change interventions: a standardised approach to evaluate intervention integrity. BMJ Open. 2013;3:e003555 10.1136/bmjopen-2012-003555 24240140 PMC3831105

[31] Moerbeek M, Wong WK. Sample size formulae for trials comparing group and individual treatments in a multilevel model. Stat Med. 2008;27:2850–2864. 17960589 10.1002/sim.3115

[32] Kahan BC, Morris TP. Assessing potential sources of clustering in individually randomised trials. BMC Med Res Methodol. 2013;13:58 10.1186/1471-2288-13-58 23590245 PMC3643875

[33] Kahan BC, Morris TP. Reporting and analysis of trials using stratified randomisation in leading medical journals: review and reanalysis. BMJ. 2012;345:e5840 10.1136/bmj.e5840 22983531 PMC3444136

[34] Kahan BC, Morris TP. Improper analysis of trials randomised using stratified blocks or minimisation. Stat Med. 2012;31:328–340. 10.1002/sim.4431 22139891

[35] Kahan BC, Morris TP. Analysis of multicentre trials with continuous outcomes: when and how should we account for centre effects? Stat Med. 2013;32:1136–1149. 10.1002/sim.5667 23112128

[36] Kahan BC, Jairath V, Doré CJ, Morris TP. The risks and rewards of covariate adjustment in randomized trials: an assessment of 12 outcomes from 8 studies. Trials. 2014;15:139 10.1186/1745-6215-15-139 24755011 PMC4022337

[37] Rubin DB. Multiple imputation for nonresponse in surveys New York: John Wiley and Sons; 1987.

[38] Carpenter JR, Goldstein H, Kenward MG. REALCOM-IMPUTE software for multilevel multiple imputation with mixed response types. J Stat Softw. 2011;45.

[39] Health and Social Care Information Centre. Secondary Uses Service (SUS). 2016 [cited 9 May 2016]. Available: http://www.hscic.gov.uk/sus.

[40] Joint Formulary Committee. British national formulary 62 edition London: BMJ Group and Pharmaceutical Press; 2011.

[41] Kennedy A, Reeves D, Bower P, Lee V, Middleton E, Richardson G, et al The effectiveness and cost effectiveness of a national lay-led self care support programme for patients with long-term conditions: a pragmatic randomised controlled trial. J Epidemiol Community Health. 2007;61:254–261. 10.1136/jech.2006.053538 17325405 PMC2652924

[42] Taylor SJC, Sohanpal R, Bremner SA, Devine A, McDaid D, Fernández J-L, et al Self-management support for moderate-to-severe chronic obstructive pulmonary disease: a pilot randomised controlled trial. Br J Gen Pract. 2012;62:e687–e695. 10.3399/bjgp12X656829 23265228 PMC3459776

[43] Claxton K, Martin S, Soares M, Rice N, Spackman E, Hinde S, et al Methods for the estimation of the National Institute for Health and Care Excellence cost-effectiveness threshold. Health Technol Assess. 2015;19:1–503. 10.3310/hta19140 PMC4781395 25692211

[44] Crawford JR, Henry JD, Crombie C, Taylor EP. Normative data for the HADS from a large non-clinical sample. Br J Clin Psychol. 2001;40:429–434. 11760618 10.1348/014466501163904

[45] Fournier JC, DeRubeis RJ, Hollon SD, Dimidjian S, Amsterdam JD, Shelton RC, et al Antidepressant drug effects and depression severity: a patient-level meta-analysis. JAMA. 2010;303:47–53. 10.1001/jama.2009.1943 20051569 PMC3712503

[46] Barth J, Munder T, Gerger H, Nüesch E, Trelle S, Znoj H, et al Comparative efficacy of seven psychotherapeutic interventions for patients with depression: a network meta-analysis. PLoS Med. 2013;10:e1001454 10.1371/journal.pmed.1001454 23723742 PMC3665892

[47] Arroll B, Elley CR, Fishman T, Goodyear‐Smith FA, Kenealy T, Blashki G, et al Antidepressants versus placebo for depression in primary care. Cochrane Database Syst Rev. 2009; 2009: CD007954 10.1002/14651858.CD007954 PMC10576545 19588448

[48] Lee H, Hubscher M, Moseley GL, Kamper SJ, Traeger AC, Mansell G, et al How does pain lead to disability? A systematic review and meta-analysis of mediation studies in people with back and neck pain. Pain. 2015;156:988–997. 10.1097/j.pain.0000000000000146 25760473

[49] Williams ACDC, Eccleston C, Morley S. Psychological therapies for the management of chronic pain (excluding headache) in adults. Cochrane Database Syst Rev. 2012; 11:CD007407 10.1002/14651858.CD007407.pub3 23152245 PMC6483325

[50] Kroenke K, Krebs EE, Wu J, Yu Z, Chumbler NR, Bair MJ. Telecare collaborative management of chronic pain in primary care: a randomized clinical trial. JAMA. 2014;312:240–248. 10.1001/jama.2014.7689 25027139

