# Supplementary material for: Novel Three-Day, Community-Based, Nonpharmacological Group Intervention for Chronic Musculoskeletal Pain (COPERS): A Randomised Clinical Trial
Source: PLoS Med. 2016 Jun 14;13(6):e1002040. doi: 10.1371/journal.pmed.1002040 (PMC4907437; doi:10.1371/journal.pmed.1002040)
Supplement: S1 Appendix — (PDF) [file pmed.1002040.s002.pdf]

Supplementary web appendices

S1. Membership of Trial Oversight Committees

S2. Full list of outcome measures

S3. Method for generating defined daily doses of prescribed psychotropic, analgesic and opioid medication

S4. Analysis of secondary outcomes

S5. Methods for sensitivity analyses for primary outcomes

S6. Deviations from the Statistical Analysis Plan V1.0 (3.10.2013)

S7. Health economics methods reported in the format suggested by the CHEERS reporting standards for trial-based evaluations

S8. Unit costs

S9. Results of sensitivity analyses

S10. Additional secondary outcomes results

S11. Results of pre-planned sub-group analyses for the primary outcome

S12 (A). Cost-effectiveness planes showing 5,000 bootstrapped estimates generated from one imputed dataset adjusted using the mixed effects linear model.

(B) Cost-effectiveness acceptability curve estimated using the incremental net benefit method from five imputed datasets adjusted using the mixed effect linear model.

Appendix S1. Membership of Trial Oversight Committees

Trial Steering Committee: Professor Mike Hurley (chair), Professor Nadine Foster,

Professor Bart Koes, Professor Lance McCracken, Mr Jim Reece, Dr Obi Ukoumunne

Data Monitoring and Ethics Committee: Professor Gene Feder (chair), Professor Blair Smith, Dr Rebecca Turner

Appendix S2. Full list of outcome measures

Primary outcome

The primary outcome is the disability subsection of the Chronic Pain Grade questionnaire (CPG disability) at 12 months post randomisation. This outcome is a composite of three questions assessing the extent to which the participant's pain has interfered with or changed their ability to perform their daily activities, work, or take part in recreational, social, and family activities in the previous six months. Each of the three questions is rated on a scale of 0-10, with 0 reflecting no change or interference, and 10 reflecting extreme change or interference.

The primary outcome is the mean of these three questions, multiplied by 10; i.e. if X1, X2, and X3 represent the three questions, and Y represents the primary outcome, then Y=10*(X1+X2+X3)/3. The primary outcome is therefore recorded on a scale from 0-100, with higher scores reflecting larger interference or change in the participant's ability to perform daily activities, work, or take part in recreational, social, and family activities.

Secondary outcomes

CPG disability at 6 months post randomisation

CPG pain intensity score at 6 and 12 months post randomisation.

PSEQ (Pain Self-Efficacy Questionnaire) score at 6 and 12 months post randomisation

HADS (Hospital Anxiety and Depression Scale) Anxiety score at 6 and 12 months post randomisation

HADS (Hospital Anxiety and Depression Scale) Depression score at 6 and 12 months post randomisation

CPAQ (Coping Pain and Acceptance Questionnaire) score at 6 and 12 months post randomisation

HEIQ (Health Education Impact Questionnaire) Social integration score at 6 and 12 months post randomisation

EQ-5D at 6 and 12 months post randomisation

Census global health question at 6 and 12 months post randomisation

Total Defined Daily Doses (Total DDD) consumed of psychotropic drugs up to 12 months post-randomisation

Total DDD consumed of analgesics (including all opioids and other CNS drugs) for pain up to 12 months post randomisation

Total DDD consumed of weak opioids up to 12 months post randomisation

Total DDD consumed of strong opioids up to 12 months post randomisation

Proportion of participants using weak opioids at 12 months post randomisation (defined as having received a prescription for a weak opioid up to twelve weeks before the 12 month follow-up date)

Proportion of participants using strong opioids at 12 months post randomisation (defined as having received a prescription for a strong opioid up to twelve weeks before the 12 month follow-up date)

Methods for deriving defined daily doses of prescribed psychotropic, analgesic and opioid medication are described in Appendix S3 (below).

Appendix S3. Method for generating defined daily doses of prescribed psychotropic, analgesic and opioid medication.

Medications prescribed over the 12 months following randomisation were collected from participants' primary care medical records. We extracted drug name and strength used, plus quantity and the dates i.e. number of times the medication was prescribed. Using the World Health Organization (WHO) defined daily dose (DDD) for each drug we generated number of days of each medication used organised by British National Formulary (BNF) chapter and subchapter [1]. The WHO does not provide DDDs for topical non-steroidal anti-inflammatory drugs (NSAIDS) or rubefaciants so we used a previously published report to define these [2].

The Total DDD for a group of medications (e.g. the Total DDD for opioids) was the sum of the Total DDD for each drug within that medication group. The DDD (used in the denominator of the calculation for the Total DDD) was determined in the first instance by the WHO register, then by precedent in other trials [2, 3] and then by clinician consensus. For compound drugs, e.g. co-codamol we separated out components (paracetamol & codeine) and worked out the DDD for each component drug i.e. paracetamol and codeine.

We considered the following outcomes:

Total Defined Daily Doses (Total DDD) consumed of psychotropic drugs (see Table below) up to 12 months post randomisation

Total DDD consumed of all analgesics up to 12 months post randomisation

Total DDD consumed of weak opioids up to 12 months post-randomisation (as defined by BNF 4.7.2 are codeine, dihydrocodeine and meptazionol)

Total DDD consumed of all NSAID analgesics (oral and topical combined) up to 12 months post randomisation

Total DDD consumed of all CNS drugs for neuropathic pain up to 12 months post-randomisation

Total DDD consumed of strong opioids up to 12 months post-randomisation (as defined by BNF 4.7.2, all opioids prescribed other than the ones listed above as weak)

Calculations for psychotropic drugs was based on BNF subchapters 4.1, and 4.3, opioids based on BNF paragraph 4.7.2, and analgesics including opioids based on BNF paragraphs 4.7.1, 4.7.2, 4.7.3, and paragraphs10.1.1, 10.2.2, and 10.3.2.

Relevant drugs

We worked out DDD for groups of drugs in BNF chapters four and 10, these are drugs used for treating chronic pain (see table below). We excluded all drugs administered as injections, but included topical preparations, patches and liquids.

Table elaborating relevant British National Formulary paragraph for included drugs.

	BNF Chapter	BNF Subchapter	BNF Paragraph with included drugs	Comments
Psychotropic drugs	4. Central Nervous System	4.1 Hypnotics & Anxiolytics	4.1.1 Hypnotics	Excluded: chloral and derivatives, clomethiazole or antihistamines
		4.3 Antidepressants	4.3.2 Monoamine-oxidase inhibitors<br>4.3.3. Selective serotonin re-uptake inhibitors<br>4.3.4 Other anti-depressant drugs	
Analgesic drugs	4. Central Nervous System	4.7 Analgesics	4.7.1 Non opioid analgesics<br>4.7.2. Opioid analgesics<br>4.7.3 Neuropathic and functional pain	4.8.1 Gabapentin and pregabalin feature as an anti-epileptic but also feature in 4.7.3 Neuropathic and functional pain<br>For this analysis 4.3.1 tricyclic anti-depressants are included in section 4.7.3
	10. Musculo-skeletal and joint diseases	10.1 Drugs used in rheumatic diseases and gout	10.1.1 Non-steroidal anti-inflammatory drugs	Excluded: aspirin, steroids, DMARDS
		10.2 Drugs used in neuromuscular disorders	10.2.2 Skeletal muscle relaxants	
		10.3 Drugs for the relief of soft tissue inflammation	10.3.2 Rubefacients and other topical anti-rheumatics	Excluded: enzymes

References

1. British National Formulary 62, September 2011. Pharmaceutical Press; 62nd edition, 2011. 1072pp

2. Underwood M, Ashby D, Carnes D, Castelnuovo E, Cross P, Harding G, Hennessy E, Letley L, Martin J, Mt-Isa S, Parsons S, Spencer A, Vickers M, Whyte K. Topical or oral ibuprofen for chronic knee pain in older people. The TOIB study. Health Technol Assess. 2008 May; 12 (22):1.

3. Underwood M, Lamb SE, Eldridge S, Sheehan B, Slowther A, Spencer A, Thorogood M, Atherton N, Bremner SA, Devine A, Diaz-Ordaz K, Ellard DR, Potter R, Spanjers K, Taylor SJ. Exercise for depression in care home residents: a randomised controlled trial with cost-effectiveness analysis (OPERA). Health Technol Assess. 2013 May;17(18):1-281. doi: 10.3310/hta17180.

Appendix S4. Analysis of secondary outcomes

CPG disability at 6 months

This outcome was analysed using the same methods as CPG disability at 12 months.

CPG pain intensity, HADS Anxiety, HADS Depression, and heiQ at 6 and 12 months

These outcomes were analysed using the same methods as CPG disability at 6 and 12 months.

PSEQ at 6 and 12 months

This outcome was specified to be analysed using the same methods as CPG disability at 6 and 12 months, except the individual components of the PSEQ score at 12 weeks will also be included in the imputation model. However, due to problems with the multiple imputation procedure, we could not run the pre-specified analysis; the analysis approach we used instead is detailed in S6.

CPAQ at 6 and 12 months

This outcome was specified to be analysed using the same methods as CPG disability at 6 and 12 months, with the exception of how CPAQ at baseline is included in the MI model. CPAQ is a composite of 20 questions – including each of these questions at each time point in the imputation model would have led to 60 variables being included (20 questions at baseline, 20 at 6 months, and 20 at 12 months) which may have caused problems. We therefore included only the individual questions for CPAQ at 6 and 12 months in the imputation model, and included the full CPAQ score at baseline (leading to 41 variables rather than 60). For participants who were missing CPAQ at baseline, we used mean imputation. However, due to problems with the multiple imputation procedure, we could not run the pre-specified analysis; the analysis approach we used instead is detailed in S6.

EQ-5D at 6 and 12 months

The EQ-5D was analysed using the same analysis model as the primary outcome (i.e. mixed-effects linear regression model, with course as a random effect, adjusted for site of recruitment, age, gender, HADS depression score, and EQ-5D at baseline).

All participants who fully completed the EQ-5D score at either 6 or 12 months were included in the analysis. EQ-5D scores with missing components were regarded as completely missing.

MI was used to account for participants who are missing the outcome at either 6 or 12 months. The MI strategy was the same as that for the primary and other secondary outcomes, except instead of imputing the individual components of the EQ-5D score, we imputed the whole score.

Census global health question at 6 and 12 months

This outcome was analysed using a mixed-effects ordered logistic regression model, with 'course' as a random effect. Site of recruitment, age, gender, HADS depression score, and the outcome at baseline were included as fixed covariates.

All participants who completed the census global health question score at either 6 or 12 months were included in the analysis.

MI was used to account for participants who are missing the outcome at either 6 or 12 months. The MI strategy was the same as that for the primary and other secondary outcomes, except we imputed the whole score (as there are no individual components).

Total DDDs up to 12 months post-randomisation for psychotropic drugs, drugs for pain, weak opioids, and strong opioids

These outcomes were analysed using a mixed-effects linear regression model, with 'course' as a random effect. Restricted maximum likelihood (REML) was used. The model included site of recruitment, age, gender, HADS depression score, and Total DDD in 3 months before randomisation at baseline as covariates. All participants who had data on Total DDD up to 12 months post-randomisation were included in the analysis. Mean imputation was used for missing baseline covariates.

Proportion of participants using weak opioids and strong opioids at 12 months post-randomisation

These outcomes were analysed using a mixed-effects logistic regression model, with 'course' as a random effect. The model included site of recruitment, age, gender, HADS depression score, and weak or strong (depending on outcome) opioid use at baseline (defined as a prescription for weak or strong) opioids in the 12 weeks before randomization) as covariates. All participants who had data on whether they had had a weak/strong opioid prescription at 12 months were included in the analysis.

Appendix S5. Methods for sensitivity analyses for primary outcomes

We performed three sensitivity analyses for the primary outcome to assess the robustness of the results to other methods of accounting for missing data[i]. The first sensitivity analysis involved specifying a different imputation model than that used in the primary analysis, and the last two sensitivity analyses involved re-analysing the primary outcome using two approaches which are not based on multiple imputation.

Sensitivity analysis 1

We determined which baseline covariates are associated with loss to follow-up, and included them in the imputation model. The analysis model was the same as above, except for the inclusion of additional covariates in the imputation model.

Sensitivity analysis 2

We performed a complete case analysis, where all participants who did not complete all components of the CPG disability score at 12 months were excluded from the analysis. The analysis model was the same as above, except missing baseline covariates were replaced using mean imputation.

Sensitivity analysis 3

We analysed the three components which form the CPG disability score at 12 months, rather than the CPG disability score itself. This was done by performing a multivariate analysis, where each of the three components from the 12 month score were included in the model as outcomes (i.e. each participant had three outcomes). A three-level mixed-effects model was used, with random effects for ‘course’ and for participant. Treatment-by-question interactions were included, allowing the treatment effect to vary for each of the three components. An overall treatment effect for CPG disability at 12 months was estimated using the lincom function in Stata to combine the treatment estimates from the three separate components. As above, missing baseline covariates were replaced using mean imputation.

Participants with no completed follow-ups

The primary analysis assumed that the excluded participants (those not completing any questions on the CPG disability questionnaire at both six and 12 months) were missing at random (i.e. they were missing based on the covariates included in the analysis model). To assess the robustness to departures from this assumption, the primary outcome was assessed under a range of missing-not-at-random scenarios. This was done using the formula $\Delta = \Delta_{primary} + Y_1P_1 - Y_2P_2$, where Δ is the treatment effect under the missing-not-at-random scenario, $\Delta_{primary}$ is the treatment effect from the primary analysis, Y_1 and Y_2 are the assumed mean responses for participants with missing data in treatment groups 1 and 2 respectively, and P_1 and P_2 are the proportion of participants who were excluded from the analysis in groups 1 and 2 respectively. The standard error for Δ is assumed to be approximately equal to the standard error for $\Delta_{primary}$. Y_2 will be varied between 10, 25, 50, 75, and 90, and for each value of Y_2, Y_1 will be set to Y_2 - 10, Y_2, and Y_2 + 10. For example, for Y_2 = 25, Y_1 will vary between 15, 25, and 35.

Reference:

i. Morris TP, Kahan BC, White IR. Choosing sensitivity analyses for randomised trials: principles. BMC medical research methodology. 2014;14(1):11.

Appendix S6. Deviations from the Statistical Analysis Plan V1.0 (3.10.2013) (One version only)

1) The mediator analyses and CACE analyse were conducted but not presented in this paper. These will be presented in a secondary / follow-up paper.

2) We did not present numbers needed to treat or relative risks for a 30% change/improvement in our primary or secondary outcomes. This change was agreed prior to any member of the trial team having access to un-blinded trial data.

3) We were unable to use our pre-specified analysis approach for PSEQ and CPAQ due to the imputation model not running:

PSEQ at 6 and 12 months

The Statistical Analysis Plan specified that the individual components of the PSEQ score at baseline, 12 weeks, 6 months, and 12 months would be imputed. However, REALCOM (the software package used for imputation) was unable to impute due to too many variables with missing data being in the imputation model.

Therefore, rather than the individual components of PSEQ at baseline, we tried to include the overall score at baseline in the imputation model (setting scores to missing if participants had any missing components). However, this did not work, as there were still too many variables with missing data in the imputation model for REALCOM to perform imputations.

Next, we tried using mean imputation to replace missing baseline scores with the overall mean of PSEQ at baseline. This allowed us to include baseline PSEQ score in the imputation model as an auxiliary variable (due to the fact it contained no missing data), rather than a variable with missing data that needed to be imputed. This method allowed REALCOM to perform imputations, and so is the basis for the analyses for PSEQ at 6 and 12 months.

CPAQ at 6 and 12 months:

The Statistical Analysis Plan specified that the individual components of the CPAQ score at 6 and 12 months would be imputed. However, REALCOM was unable to impute due to too many variables with missing data being in the imputation model.

In order to reduce the total number of variables, we combined the individual components into pairs, leading to 10 pairs of two at each time point. For example, if Q1, Q2, …, Q20 are the 20 individual questions that form the overall CPAQ score at any time point, we generated 10 pairs as:

P1 = Q1 + Q2, P2 = Q3 + Q4, …, P10 = Q19 + Q20

We set P to missing if either of the Qs involved were missing. We then included the Ps in the imputation model at 6 and 12 months, reducing the total number of variables from 40 to 20. This method allowed REALCOM to perform imputations, and so is the basis for the analyses for CPAQ at 6 and 12 months.

Appendix S7. Health economics methods reported in the format suggested by the CHEERS reporting standards for trial-based evaluations1

Target population	The target population were adults with chronic musculoskeletal pain, analysed on an intention to treat basis.
Setting and location	Primary care and musculoskeletal physiotherapy services in two localities; east London and Coventry/Warwickshire
Study perspective comparators	The comparator was usual care
Time horizon	12 months
Discount rate	1
Choice of health outcomes measurement of effectiveness	Health-related Quality of life measured using EQ-5D 3L and Quality-adjusted life years
Measurement and valuation of preference-based outcomes estimating resources and costs	Valuation: The EQ-5D 3L was converted to Quality Adjusted Life Years (QALYs) by using the UK tariff based on the time trade off method2.<br><br>Resource Use: We extracted data from the NHS electronic records, including the Secondary Uses Service (SUS) database and GP records.<br><br>Units costs: See more details in appendix S8.
Currency, price date, and conversion	2011-2013, GBP, costs were converted to USD using purchasing power parity rate
Analytic methods	We used mixed effects regression model with intervention group as a random effect. Sensitivity analyses were conducted using a generalized linear model (GLM), and a seemingly unrelated regression (SUR) model. Uncertainty associated with costs and outcomes was addressed by calculating cost effectiveness acceptability curves using the three models (MLM, GLM and SUR).<br><br>Missing data was imputed using the predictive mean matching imputation method, which has been recommended for skewed distributions2. Covariates included in the imputation were age, gender, site of recruitment (London or Midlands), course, and HADS depression scores at baseline. Multiple imputations were conducted for primary and secondary care costs (12 months post-randomisation), baseline prescriptions (three months pre-randomisation), and the EQ-5D (baseline, six and 12 month post-randomisation). Results from the imputation were combined using Rubin's rule. In the imputed data set a parametric approach to address the uncertainty around ICER point estimates.

1. Husereau D, Drummond M, Petrou S, Carswell C, Moher D et al. ISPOR Health Economic Evaluation Publication Guidelines-CHEERS Good Reporting Practices Task Force. Consolidated Health Economic Evaluation Reporting Standards (CHEERS) - explanation and elaboration: a report of the ISPOR Health Economic Evaluation Publication Guidelines Good Reporting Practices Task Force. 2013 Value Health;16:231-50.

2 Dolan P. Modeling valuations for EuroQol health states. Med Care 1997; 3:1095-108.

3 Little, RJA. 1988. Missing-data adjustments in large surveys. Journal of Business and Economic Statistics;6:287-96.

Appendix S8. Unit costs

Individual-level resource use data were combined with unit costs to calculate the total cost of health service use for each participant. Primary care consultations and referrals to community care were costed using the Unit Costs of Health and Social Care 2012.[1] Unit costs which were not available in this source were supplemented with costs from the National Schedule of Reference Costs 2011-2012.[2] Tests and investigations were costed using the National Schedule of Reference Costs 2011-2012, [2] direct Access Diagnostic and Pathology Services. Prescriptions were analysed using the Prescription Cost Analysis (PCA) database 2011-2012. [3,4] If costs were missing in the BNF, we checked them in the NHS Drug Tariff, [5] MIMS, [6] or in the retailers' price lists (for items other than drugs). Items missing in all the above sources were substituted with alternative items from the PCA database, which contain the same active ingredients/strengths.

The costs of secondary health care services used by participants were downloaded as a part of the SUS database. Where costs were not provided, the National Schedule of Reference Costs 2011-20122 were used. Outpatient costs were matched by specialty code. The average unit costs (all NHS trusts) were used given insufficient information about the type of outpatient appointment (consultant-led/non-consultant led, face-to-face/non-face-to face) provided by SUS. Inpatient and A&E costs were matched by the health resource group (HRG) code. The average HRG costs (all NHS trusts) were used due to lack of information about inpatient stay (elective/non-elective, short stay/long stay) provided by SUS. A&E costs were assumed to be 'Not Leading to Admitted'.

References

1. Personal Social Services Research Unit (PSSRU). Unit Costs of Health and Social Care 2012. Available at http://www.pssru.ac.uk/project-pages/unit-costs/2012/ [Accessed 1st September 2013]

2. Department of Health. National Schedule of Reference Costs 2011-2012 - NHS trusts and NHS foundation trusts. Available at https://www.gov.uk/government/publications/nhs-reference-costs-financial-year-2011-to-2012 [Accessed 1st September 2013]

3. NHS Business Services Authority. Prescription Cost Analysis. Available at http://www.nhsbsa.nhs.uk/PrescriptionServices/3494.aspx [Accessed 1st September 2013]

4. British National Formulary (BNF) Available at http://www.bnf.org/bnf/ [Accessed 1st September 2013]

5. NHS Business Services Authority. Drug Tariff. August 2013, Available at http://www.nhsbsa.nhs.uk/924.aspx [Accessed 1st September 2013]

6. Monthly Index of Medical Specialities (MIMS). Available at http://www.mims.co.uk/ [Accessed 1st September 2013]

Appendix S9: Results of sensitivity analyses

All sensitivity analyses found similar results to the primary analysis anddemonstrated that primary outcome results were robust (see Table and Figure below).

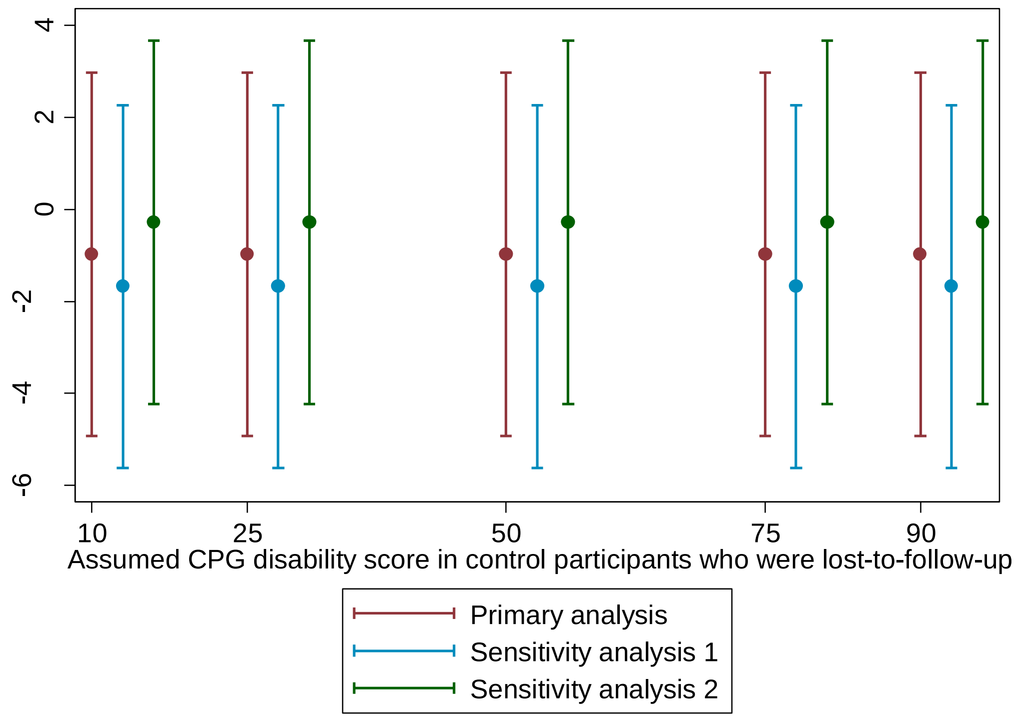

Figure: Sensitivity analysis for CPG disability at 12 months (assuming missing data are missing-not-at-random)

The Y-axis in the Figure above shows the treatment effect for the CPG disability score at 12 months (e.g. a value of -2 indicates that the mean CPG disability score was two points less in the intervention group than in the control group). The X-axis shows the assumed CPG disability score in participants in the control group who were lost to follow-up (e.g. a value of 10 indicates that we set the average CPG disability score for participants in the control arm who were lost to follow-up to 10). Sensitivity analysis 1 set the CPG disability score for participants in the intervention arm who were lost to follow-up to 10 points less than participants in the control arm (e.g. if the value on the X-axis was 10, this would indicate that participants in the control arm who were lost to follow-up had a CPG disability score of 10, and participants in the intervention arm who were lost to follow-up had a CPG disability score of 0). Sensitivity analysis 2 set the CPG disability score for participants in the intervention arm who were lost to follow-up to 10 points higher than participants in the control arm (e.g. if the value on the X-axis was 10, this would indicate that participants in the control arm who were lost to follow-up had a CPG disability score of 10, and participants in the intervention arm who were lost to follow-up had a CPG disability score of 20).

The primary analysis excluded all participants who did not complete any CPG disability questions at either six or 12 months. That analysis assumed the excluded participants were missing-at-random – that is, the reason these participant's data was missing was based upon variables that were included in the analysis (e.g. that older participants with high baseline CPG disability scores were more likely to be excluded from the analysis).

The analyses in the Figure have made different assumptions regarding participants who were excluded from the analysis in order to assess how robust the primary analysis results are to departures from the missing-at-random assumption. Specifically, the analyses in the Figure have assumed the excluded participants were missing-not-at-random – that is, the reason these participant's data was missing was actually based upon their CPG disability scores at six and 12 months (e.g. participants with higher CPG disability scores at six and 12 months were more likely to be excluded from the analysis).

The results in the Figure indicate that results from the primary analysis for CPG disability are robust to departures from the missing-at-random assumption (i.e. even if the missing data from participants who were lost to follow-up was missing-not-at-random, this would not alter the conclusions from our main analysis).

Table: Sensitivity analyses for CPG disability at 12 months

	Treatment effect (95% CI)
Main analysis	-1.0 (-4.9 to 3.0)
Complete case analysis	-0.9 (-4.9 to 3.1)
Multivariate analysis	-0.1 (-5.5 to 5.2)
Different imputation model	-1.1 (-5.1 to 2.9)
CACE analysis	-1.0 (-5.9 to 3.9)

Appendix S10. Additional secondary outcomes results

Census Global Health Questionnaire

There was no difference in responses to the Census global health question detected between the intervention and control arms at either 6 or 12 months follow up (odds ratio, OR, for intervention group participants being in a higher category at 6 months was 1.09, 95% CI 0.77 to 1.54; OR at 12 months 1.07, 95% CI 0.77 to 1.51).The table below presents results based on available data; those not providing data are excluded.

Table: Responses to census global health question, N (%), at baseline six and 12 months follow-up

Census global health question	BaselineN (%)		Six MonthsN (%)		12 monthsN (%)	
	Control	Interven-tion	Control	Interven-tion	Control	Interven-tion
Very good	17 (6)	27 (7)	11 (5)	20 (6)	8 (3)	14 (4)
Good	100 (33)	138 (34)	81 (34)	121 (35)	84 (34)	130 (38)
Fair	130 (43)	159 (39)	100 (42)	144 (42)	115 (47)	144 (42)
Bad	45 (15)	63 (16)	39 (16)	46 (13)	32 (13)	40 (12)
Very bad	8 (3)	16 (4)	7 (3)	11 (3)	6 (2)	14 (4)
Totals	300 (100)	403 (100)	238 (100)	342 (100)	245 (100)	342 (100)

Prescribed Medicines

Differences in prescribed medicines, expressed as Defined Daily Dose (DDD), in the treatment arms at 12 months follow-up are presented in the table below. Intervention arm patients were prescribed significantly more DDDs of weak opioids in the 12 months following randomisation than those in the control arm amounting to a difference of 18 days of medication at WHO standard dosing, (95% CI 5 to 32 days). The proportion of intervention arm participants taking weak opioids at 12 months also tended to be higher in the intervention group, although the difference was not statistically significant (the odds of taking weak opioids was increased by 39% in intervention arm, 95% CI 10 % fewer to 114% more).

Overall intervention patients received considerably more analgesics than control arm patients in the 12 months after randomisation (98 DDDs, 95% CI 17 to 178). However there was no evidence of any difference in the prescription of strong opioids between treatment arms (-1 DDD 95% CI -12 to 11) nor in the proportions of those receiving strong opioids at 12 months (the odds of taking strong opioids was increased by 4% in intervention arm, 95% CI 41% fewer to 85% more).

Table: Total amount of drugs prescribed as Defined Daily Dose (DDD) in the 12 months post-randomisation and proportion of participants using opioids at 12 months post-randomisation*

	Control (n=258*)	Intervention (n=350*)	Treatment effecta (95% CI)
DDD in 12 months post-randomisation, median (IQR)			
Psychotropics	0 (0 to 21)	0 (0 to 28)	-12 (-30 to 6)
Weak opioids	0 (0 to 36)	0 (0 to 64)	18 (5 to 32)
Strong opioids	0 (0 to 22)	0 (0 to 24)	-1 (-12 to 11)
Analgesics (including opioids and other CNS drugs)	232 (45 to 551)	295 (57 to 648)	98 (17 to 178)
Proportion of participants using opioids at 12 months post-randomisation – no. (%)			
Weak opioids	59 (23)	103 (29)	1.39 (0.90 to 2.14)
Strong opioids	64 (25)*	82 (23)*	1.04 (0.59 to 1.85)

*258 participants (86%) in the control arm, and 350 (87%) participants in the intervention arm had drug prescription data available and were included in the analysis.

aTreatment effect represents a difference in means for DDD outcomes, and an odds ratio for opioid use outcomes at 12 months

Appendix S11. Results of pre-planned sub-group analyses for the primary outcome

The results of our pre-planned sub-group analyses for the primary outcome of CPG pain related disability subscale at 12 months are presented in the table below. There is no evidence to support the intervention being more effective in those who live alone, or have four or more co-morbidities or have lower socioeconomic status.

There is the suggestion of a non-significant tendency for those with shorter pain duration to show more benefit in terms of the primary outcome - however interpretation is difficult as this sub-group analysis is hampered by small numbers since the vast majority of participants had longstanding pain. There was no evidence that treatment effects differed across sub-groups.

No trend is seen in the association between pain related self-efficacy and primary outcome however there is an (inconclusive) suggestion that the effect size might be greatest in the group with an intermediate level of baseline self-efficacy.

Finally, there is a suggestion that those with HADS depression subscale scores highly indicative of the likelihood of depression (scores of 11 or more) may have shown much greater improvement in pain related disability at 12 months but again, the numbers are relatively small and this finding is not statistically significant.

Table: Results of pre-planned sub-group analyses for primary outcome (CPG disability at 12 months)						
	Number included in analysis					
Sub-group	Control	Intervention	Control, mean (SD)	Intervention, mean (SD)	Treatment effect (95% CI)	P value for interaction
Co-morbidity						
0-3	192	269	50.2 (29.2)	50.6 (27.7)	-0.6 (-5.1 to 4.0)	0.72
4 or more	76	90	59.8 (26.8)	57.8 (28.0)	-2.1 (-9.4 to 5.3)	
Living arrangements						
Living with others	185	239	52.4 (28.1)	50.9 (28.0)	-0.1 (-4.9 to 4.8)	0.60
Living alone	89	129	54.5 (30.8)	56.9 (27.4)	-2.2 (-8.9 to 4.5)	
PSEQ						
0-20	72	83	71.7 (22.5)	72.8 (23.6)	0.5 (-7.0 to 7.9)	0.78
21-39	121	184	56.5 (23.2)	54.6 (24.2)	-2.2 (-7.6 to 3.3)	
40-60	85	103	34.0 (29.1)	34.6 (25.9)	0.4 (-6.4 to 7.1)	
Socioeconomic status						
Lower	136	197	52.0 (29.3)	48.5 (27.3)	-2.4 (-7.8 to 3.0)	0.42
Higher	142	177	54.6 (28.4)	57.9 (28.1)	0.8 (-4.7 to 6.2)	
Pain duration						
0-12 months	13	13	40.0 (30.3)	31.8 (29.4)	-5.5 (-23.5 to 12.6)	0.88
13 months to 4yrs	80	93	51.7 (29.2)	51.3 (26.6)	-1.7 (-8.9 to 5.4)	
5 or more yrs	185	268	54.9 (28.5)	54.5 (28.1)	-0.8 (-5.5 to 3.8)	

**Table: Results of pre-planned sub-group analyses for primary outcome (CPG disability at 12 months) (Contd.)**						
	Number included in analysis					
Sub-group	Sub-group	Sub-group	Sub-group	Sub-group	Sub-group	Sub-group
CPG pain intensity						
0-3	4	17	45.0 (42.6)	21.6 (20.1)	-22.5 (-47.9 to 2.8)	0.24
4-7	186	219	47.1 (28.1)	46.1 (25.8)	-1.0 (-5.8 to 3.8)	
8-10	87	138	66.7 (25.3)	67.4 (25.1)	-0.2 (-6.6 to 6.3)	
CPG disability						
0-3	51	70	31.7 (27.6)	33.3 (27.4)	0.5 (-8.1 to 9.1)	0.60
4-7	138	187	51.1 (26.1)	48.4 (23.7)	-2.8 (-8.2 to 2.5)	
8-10	89	117	69.8 (23.8)	71.5 (23.6)	1.1 (-5.6 to 7.8)	
HADS depression score						
0-10	222	291	49.0 (28.7)	49.0 (27.4)	-0.2 (-4.6 to 4.2)	0.44
11-21	56	83	70.6 (22.5)	67.1 (25.7)	-3.8 (-12.0 to 4.4)	

a Participants with missing baseline values of the sub-group were excluded from the analysis, for all subgroup analyses apart from CPG disability and HADS depression score.

b Note – the numbers included in each subgroup are approximate for CPG disability and HADS depression score, as these variables were included in the multiple imputation model. Therefore, participants with a missing baseline value of CPG disability or HADS depression score were included in the analysis, however it is unclear which of the sub-groups they belong

Appendix S12

(A). Cost-effectiveness planes showing 5,000 bootstrapped estimates generated from one imputed dataset adjusted using the mixed effects linear model.

(B) Cost-effectiveness acceptability curve estimated using the incremental net benefit method from five imputed datasets adjusted using the mixed effect linear model.

(A)

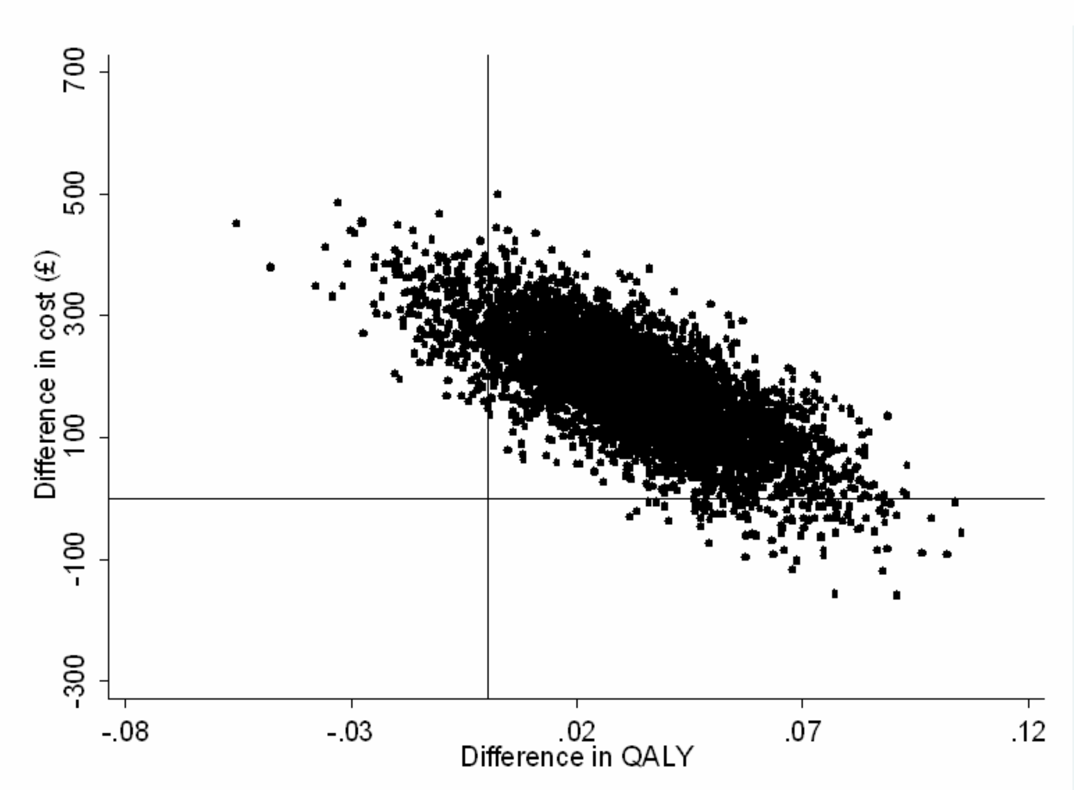

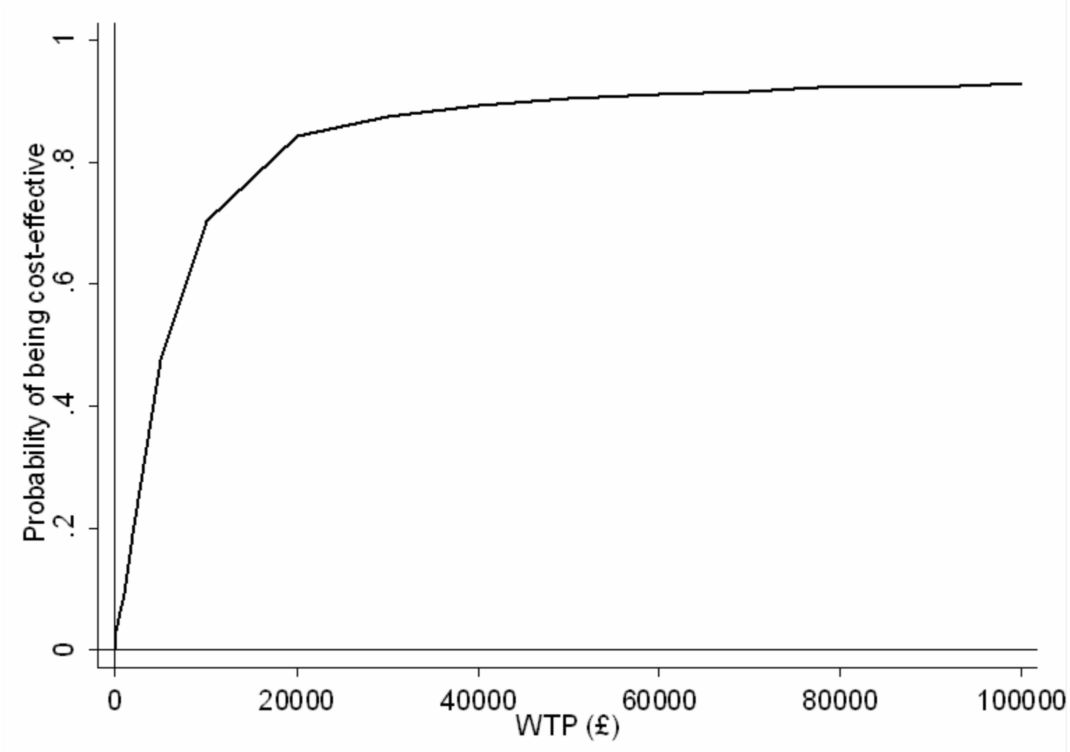

Probability of being cost-effective
0
.2
.4
.6
.8
1
0
20000
40000
60000
80000
100000
WTP (£)

(B)
