# Supplementary material for: Novel Three-Day, Community-Based, Nonpharmacological Group Intervention for Chronic Musculoskeletal Pain (COPERS): A Randomised Clinical Trial
Source: PLoS Med. 2016 Jun 14;13(6):e1002040. doi: 10.1371/journal.pmed.1002040 (PMC4907437; doi:10.1371/journal.pmed.1002040)
Supplement: S1 Protocol — (PDF) [file pmed.1002040.s004.pdf]

Barts and The London NHS
NHS Trust

Barts and The London
School of Medicine and Dentistry

# COping with persistent Pain, Effectiveness Research for Self-management: a randomised controlled trial

**Short title/Acronym:** **COPERS Trial**

**Sponsor:** Barts and The London NHS Trust/ Barts and The London School of Medicine and Dentistry

Representative of the Sponsor:
**Gerry Leonard**
**Head of Resources**
Joint R&D Office
5 Walden Street
London
E1 2EF
Phone: 020 7882 7260
Email: gerry.leonard@bartsandthelondon.nhs.uk

**REC reference:** **11/EE/0046**
**ISRCTN:** **24426731**

## Chief Investigator Agreement Page

The clinical study as detailed within this research protocol **(Version 10, 10.1.11)**, or any subsequent amendments will be conducted in accordance with the Research Governance Framework for Health & Social Care (2005), the World Medical Association Declaration of Helsinki (1996) and the current applicable regulatory requirements and any subsequent amendments of the appropriate regulations.

**Chief Investigator Names:**

**1) Professor Stephanie Taylor, Site 1: Barts and The London School of Medicine and Dentistry, Queen Mary University of London**

**Signature and Date:**

**2) Professor Martin Underwood, Site 2: Warwick Medical School, Coventry**

**Signature and Date:**

**Statistician Agreement Page**

The clinical study as detailed within this research protocol **(Version 10,10.1.11)**, or any subsequent amendments will be conducted in accordance with the Research Governance Framework for Health & Social Care (2005), the World Medical Association Declaration of Helsinki (1996) and the current applicable regulatory requirements and any subsequent amendments of the appropriate regulations.

**Statistician Name: Professor Sandra Eldridge**

**Site: Barts and The London School of Medicine and Dentistry, Queen Mary University of London**

**Signature and Date:**

**Principal Investigator / Study Manager Agreement Page**

The clinical study as detailed within this research protocol **(Version10 10.1.11)**, or any subsequent amendments will be conducted in accordance with the Research Governance Framework for Health & Social Care (2005), the World Medical Association Declaration of Helsinki (1996) and the current applicable regulatory requirements and any subsequent amendments of the appropriate regulations.

**Principal Investigator/ Study Manager Name: Dr Dawn Carnes**

**Principal Investigator Site: Barts and The London School of Medicine and Dentistry, Queen Mary University of London**

**Signature and Date:**

## STUDY SUMMARY/SYNOPSIS

| | |
|---|---|
| **TITLE** | **CO**ping with persistent **P**ain, **E**ffectiveness **R**esearch for **S**elf-management: a randomised controlled trial |
| **SHORT TITLE** | COPERS Trial |
| **Protocol Version Number and Date** | V11 7.9.11 |
| **Methodology** | Randomised controlled trial |
| **Study Duration** | Estimated duration January 2011 to January 2014 |
| **Study Centres** | Barts and The London School of Medicine and Dentistry, Queen Mary University of London and Warwick Medical School, Coventry |
| **Objectives** | To test the effectiveness and cost effectiveness of a group self-management course for people with persistent pain |
| **Number of Subjects/Patients** | 700 |
| **Main Inclusion Criteria** | Adults with chronic musculoskeletal pain |
| **Statistical Methodology and Analysis** | To estimate treatment effects we envisage using a linear mixed effects model adjusted for age, gender, centre (Tower Hamlets or Warwickshire) and baseline value of outcome. We aim to report the standardised mean differences, numbers needed to treat (NNTs) and relative risks for a 30% change/improvement for our primary and secondary outcomes. |

## Glossary of Terms and Abbreviations

| | |
|---|---|
| AE | Adverse Event |
| AR | Adverse Reaction |
| ASR | Annual Safety Report |
| CA | Competent Authority |
| CI | Chief Investigator |
| CRF | Case Report Form |
| CRO | Contract Research Organisation |
| DMC | Data Monitoring Committee |
| EC | European Commission |
| GAfREC | Governance Arrangements for NHS Research Ethics Committees |
| ICF | Informed Consent Form |
| ISRCTN | International Standard Randomised Controlled Trial Number |
| JRO | Joint Research and Development Office |
| MA | Marketing Authorisation |
| MS | Member State |
| Main REC | Main Research Ethics Committee |
| NHS R&D | National Health Service Research & Development |
| PI | Principle Investigator |
| QA | Quality Assurance |
| QC | Quality Control |
| Participant | An individual who takes part in a clinical trial |
| RCT | Randomised Controlled Trial |
| REC | Research Ethics Committee |
| SAE | Serious Adverse Event |
| SDV | Source Document Verification |
| SOP | Standard Operating Procedure |
| SSA | Site Specific Assessment |
| TMG | Trial Management Group |
| TSC | Trial Steering Committee |

Index

**1. Introduction**
1.1 Background
1.2 Rationale and Risks/Benefits

**2. Trial Objectives and Design**
2.1 Trial Objectives
2.2 Trial Design
2.3 Study Scheme Diagram

**3. Subject Selection and recruitment**
3.1 Recruitment of recruiting sites
3.2 Number of Subjects and Subject Selection
3.3 Inclusion Criteria
3.4 Exclusion Criteria

**4. The intervention and the control**
4.1 The intervention
4.2 Facilities
4.3 Recruiting facilitators and facilitator training
4.4 Quality control and intervention treatment drift
4.5 The control

**5. Study Procedures**
5.1 Informed Consent Procedures
5.2 Screening Procedures
5.3 Randomisation Procedures
5.4 Schedule of Treatment
5.5 Schedule of Assessment
5.6 Follow up Procedures
5.7 End of Study Definition
5.8 Subject Withdrawal
5.9 Data Collection and Follow-up

**6. Adverse events vigilance**

6.1 General Definitions
6.1.1 Adverse Event (AE)
6.1.2 Serious Adverse Event (SAE)
6.1.3 Other adverse events
6.2 Notification and reporting Adverse Events or Reactions
6.3 Annual Safety Reporting
6.4 General Safety Issues

**7. Statistical Considerations**
7.1 Primary Outcomes
7.2 Secondary Outcomes
7.3 Sample Size
7.4 Statistical Analysis

**8. Data Handling & Record Keeping**
8.1 Confidentiality
8.2 Study Documents
8.3 Case Report Form

8.4 Record Retention and Archiving
8.5 Compliance
8.6 Clinical Governance Issues
8.6.1 Ethical Considerations
8.7 Quality Control and Quality Assurance
8.7.1 Summary Monitoring Plan
8.7.2 Audit and Inspection
8.8 Non-Compliance

**9. Trial Committees**

**10. Publication Policy**

**11. References**

**12. Appendices**

## 1. Introduction

### 1.1 Background

Chronic conditions, especially musculoskeletal conditions, impose an increasing burden on the NHS and on society (Maniakadis and Gray, 2000). In 2009, the Chief Medical Officer highlighted chronic pain as an issue that needs addressing (DH Policy Paper 2004). Estimates of the prevalence of chronic musculoskeletal pain range from 46% to 76% (Parsons et al, 2007). Despite increased understanding of the factors contributing to the development of chronic pain, there has been little improvement in how successfully it is managed (Croft 2000).
A key component of the Department of Health (DH) response to the growing burden of chronic disease has been to promote self-management (Secretary of State for Health 2001) through the introduction and promotion of its flagship, lay-led (ie peer-led), self-management training course, the Expert Patients Programme (EPP) (EPP website). The EPP is a six-week course of one group session (3 hours) per week, usually with around 10 -16 participants. The DH decided that the EPP would be "mainstreamed" within the NHS by 2008 (Wanless 2002). In practice, the uptake of the EPP appears to have been slower and patchier than the DH had originally anticipated, although they had invested some £18 million in the EPP by 2007 (Prime Minister speech 2008).

The national evaluation of the original EPP reported a significant increase in patients' self-efficacy (confidence), with a standardised effect size of 0.4, and self reported energy levels (effect size 0.18), but no reduction in health care utilization (Kennedy et al, 2007). Our Cochrane review of the effectiveness of lay-led self-management programmes for chronic conditions suggested that the beneficial effects are modest in the short term and demonstrated a paucity of evidence on long-term benefit (Foster et al, 2007).

Concern about the efficacy of the original EPP and the impetus to provide more effective self management for patients with chronic pain has enabled us to obtain a five-year National Institute of Health Research (NIHR) funded programme grant to explore aspects of current self-management initiatives that are effective and to identify factors that determine which patients do well and which ones don't with these types of interventions with a view to developing, or refining, and formally evaluating a new intervention.

During 2009 we systematically reviewed the evidence for self-management courses for chronic pain; considering their component parts; predictors of outcome; and moderators and mediators of successful outcomes following self management interventions. We also conducted extensive research to assess the most appropriate outcome measurement tools to assess our new intervention. Additionally, we conducted a qualitative study to explore views and opinions about expectations, course content, recruitment, tutoring and attendance at self-management courses for chronic pain.

Our research to date has enabled us to develop and pilot a new self-management course. It includes: psychological (cognitive behavioural approaches), pain education and activity components. It is delivered in a group setting at venues local to participants. . The course is joint lay and health care professional led. Our systematic review found that the length of the courses (either under or over eight weeks) had no bearing on outcome (Carnes et al 2011). The course is run over three short days in one week with a 2 hour follow-up session 2 weeks later. The most influential

predictor on outcome for self-management courses from our systematic reviews was self-efficacy.
The pilot study indicated that the course was feasible and well received by participants. We used the pilot trial participant feedback questionnaires, 14 participant interviews, facilitator reflections and focus group and course observations to modify the intervention. At each stage of the pilot trial chronic pain patients have been consulted. Not only did they contribute to enhancing the delivery and content of the intervention, but they also commented on trial procedures and materials.

### 1.2 Rationale and Risks/Benefits

The next phase of this research programme is to test the effectiveness and cost effectiveness of the newly designed course in a randomised controlled trial. This study will help determine whether it is worth the Department of Health funding similar interventions and, if so, how they should be constructed and delivered. We know at present the effectiveness of EPP in terms of NHS cost saving is negligible (Kennedy et al 2007), the benefit of exploring effectiveness and cost effectiveness of the next generation of these self-management programmes will show whether further investment is appropriate or not.

## 2. Trial Objectives and Design

### 2.1 Trial Objective

To test the effectiveness and cost-effectiveness of a self-management course for chronic pain

- *Primary Outcomes*
  Effectiveness - Pain related disability
  Health Economics - Incremental Cost Utility Ratio (ICUR).
- *Secondary Outcomes*
  Coping skills, anxiety, depression, social integration and self-efficacy.

### 2.2 Trial Design

Unmasked pragmatic randomised controlled trial to test the effectiveness of a self-management course for chronic pain against a control consisting of usual GP care, a patient education leaflet and a relaxation CD.

Two study centres will be used, one in London (Tower Hamlets, City and Hackney, Newham), the other in Warwickshire (Warwick and Coventry)

2.3 Study Scheme Diagram

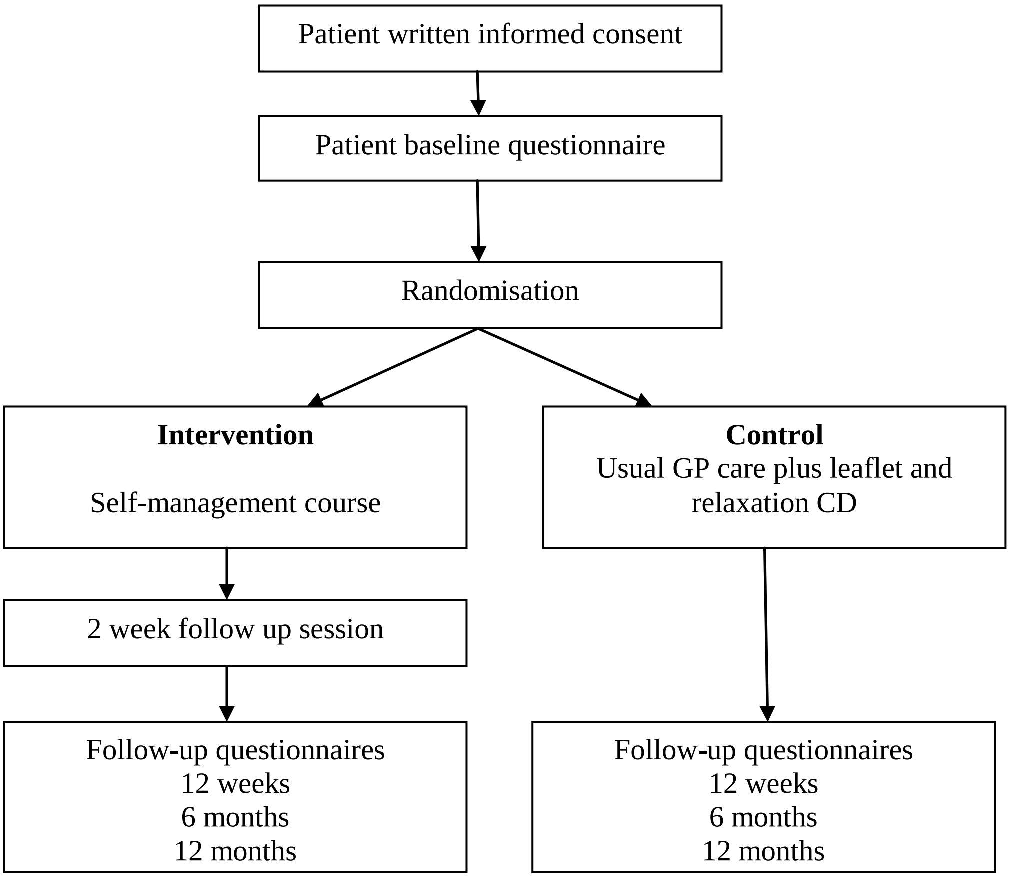

## 3. Subject Selection and recruitment

3.1 Recruitment of recruiting centres
General practices, pain clinics and musculoskeletal physiotherapy services will be recruited with the assistance of the primary care research networks (PCRNs) in London and the Midlands and our own peer networks. All general practices in the trusts involved will be invited to participate, the study team and the PCRNs will approach the general practices, those expressing an interest will be approached via the study team and if after further enquiry the general practices are in still interested, they will be asked to sign a study agreement form and re-imbursement of costs will be formalised. Service support costs will be sought via the CLRN.

The London PCRN feasibility advocate will help determine the service support required. They will pilot the search process in one GP clinic to determine the level of expertise, resources and time required and help us calculate the service support costs involved.

3.2 Number of Subjects and Subject Selection
Our target population is adults (persons aged over 18 years) living with chronic musculoskeletal pain (see inclusion criteria below for definitions used). Patients will be recruited from primary care practices, NHS musculoskeletal physiotherapy units and NHS pain clinics.

**Recruitment flow chart**

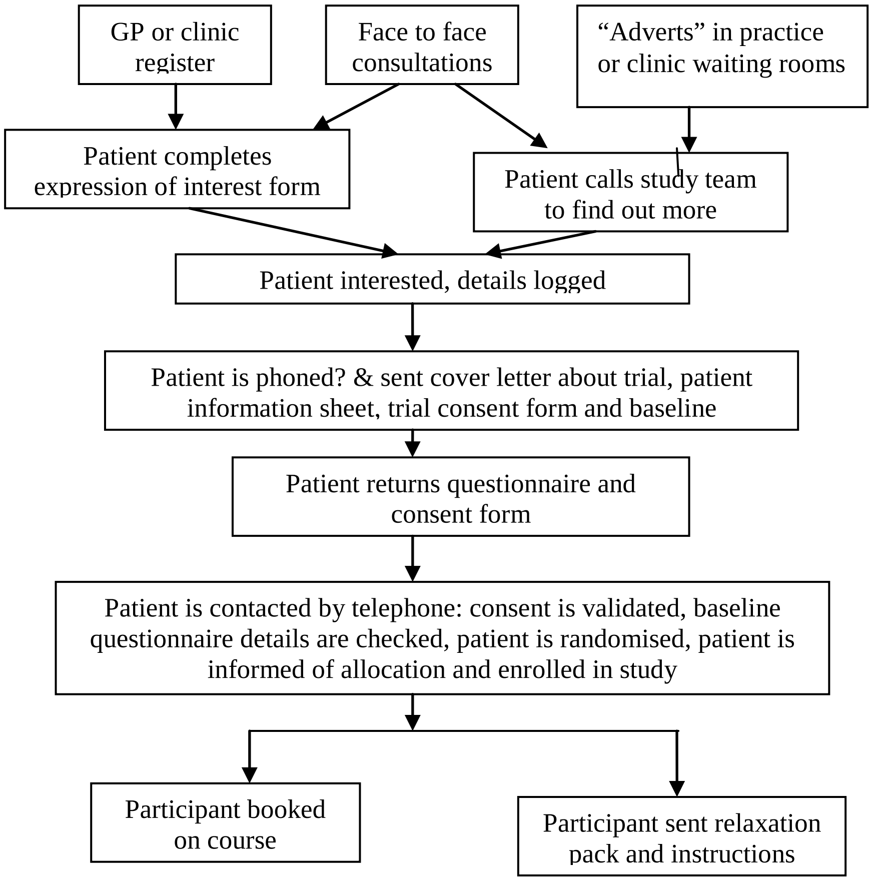


Participants will be recruited in three ways:

a. Electronic searches using the clinic data bases
b. GP/clinician referrals during face to face consultations
c. Advertisements in clinics

The electronic searches will be conducted by clinic staff with the support of a Primary Care Research Network (PCRN) research officer and / or the COPERS research study team pending appropriate NHS approvals.

We have tested a search strategy to identify the most appropriate patients using general practitioners' electronic patient registers. A general practice staff member conducted several searches of the practice electronic records to identify the most appropriate domains and search terms, these search results were reviewed by a clinician in the practice to check the appropriateness of the sample. Two people then independently searched the clinic records electronically using the same search instructions to test the reliability of the output and the search method and subsequent validity.

The first stage of the search is to identify registered patients who have consulted within the last 3 months, then within this group the second stage is to search for prescribing information about repeat prescriptions for antidepressants medication, hypnotics and analgesia. Finally we search by symptoms: low back pain, back ache, musculoskeletal, connective tissue disorders and pain. This will generate a list of potential participants. Each clinic will designate a key contact to liaise with the PCRN

and the study team; these personnel will be trained to conduct their own searches by the study team and will be given a study manual outlining the standard protocols necessary for the study. They will be given support and advice as required.

From previous searches and test runs we estimate that this type of search will yield around 5% of the registered patients, which supports other epidemiological research estimates that 5 – 10% of the population experience chronic pain.

A list of potential participants will be produced, these will be screened by the clinicians to check suitability; no vulnerable people will be approached (see inclusion and exclusion criteria below). The study team will be provided with a pooled anonymous data set to allow response rates to be calculated. This list will contain, gender, age (not date of birth) and ethnicity (if recorded). Once the list has been finalised the study representative will print off invitation letters from the patient's GP or clinician. These will be placed in pre-prepared envelopes that will contain, the 'consent to approach' form, a patient information leaflet and a freepost envelope to return the 'consent to approach' forms to the study team. There will be a single postal reminder after 10-14 days. Any interested patients will be able to complete a 'consent to approach' form and send this to the study team in a freepost envelope, or they can phone or email the study team directly to express interest and find out more about the study. Those who find out about the study via the waiting room advertisements will contact the study team directly or pick up an invitation pack from the GP receptionists. In these cases the study team will screen and check suitability to participate by using the inclusion and exclusion criteria as a checklist. GPs and clinicians will be informed of all their patients enrolled into the study but they will not be informed of their allocation.
All patients interested in taking part in the study will be sent a COPERS invitation cover letter, patient information leaflet, a baseline questionnaire, a trial consent form and a freepost return envelope.

### 3.2 Inclusion Criteria

Adults (aged 18 or over) with chronic musculoskeletal pain.

The International Association for the Study of Pain (IASP) defines chronic pain as that which has persisted beyond normal tissue healing time - usually interpreted as three months (IASP 1986). Examples include osteoarthritis, any chronic musculoskeletal pain, chronic widespread pain and fibromyalgia; we will exclude inflammatory arthritis such as rheumatoid arthritis.
We will also exclude chronic pain arising from malignant disease because it requires specific management. However, we recognise that chronic pain in patients with cancer, or those who have survived cancer, may arise from non-malignant causes and such patients would be eligible for our studies.

### 3.3 Exclusion Criteria

Not fluent in English.
Serious active co-morbidity that is more disabling to the individual than chronic pain,
Serious mental health issues that would make it difficult for an individual to participate in the group course.
Patients with a life expectancy of less than six months.
Substance misuse that would make it difficult for an individual to participate in the group course.
Inability to give informed consent.

We have restricted the study to those who are fluent in spoken English for three practical reasons.  Firstly the interactive group nature of the intervention means that it is not suitable to be delivered through an interpreter.  Secondly, in our systematic review we identified lack of fluency in the language of the programme as to be associated with lack of clinical effect.  Thirdly, the only other language that is sufficiently common to consider running courses in the areas in which the trial will be conducted is Sylheti. We have piloted delivering the intervention in Sylheti and have found that the operational issues made it impractical to include multiple language streams for the intervention.  Additionally the validity and reliability of the outcome measures when used in languages other than English has not always been established meaning that there would be concerns about quality for data collected from those who are not fluent in English.

3.4 Criteria for Withdrawal

All participants will be free to withdraw from the study at any time and without having to give any explanation, upon formal withdrawal from the study we will cease to collect further data.

**4. The Intervention and the Control**

4.1 The intervention

The intervention is a group based, facilitated learning course about chronic pain. The course is led by two facilitators, a health care professional (physiotherapist, osteopath, chiropractor, occupational health practitioner or psychologist) and a lay person with chronic pain and prior experience of small group facilitation though involvement as an Expert patient Programme tutor. All facilitators have training.
We aim to have around 12 participants per course. The minimum required for a course to take place is 8 people and the maximum is 16. Should a course be undersubscribed those registered will be offered alternative dates for other courses. The course will cover various aspects of pain education and pain management. It will be run over three days within one week with a two hour follow up session after two weeks (table 1).

**Table 1**

**Course overview**

| Day | Modules | Content of sessions |
|---|---|---|
| 1<br>Living and dealing with pain | 1. Introduction and Understanding pain and acceptance | Session 1: Introduction<br>Session 2: Pain information<br>Session 3: Acceptance: The uninvited guest |
| | Lunch | |
| | Taster activity | Art |
| | 2. Mind, mood and pain | Session 4: Pain, when is it bearable and when is it not?<br>Session 5: The pain cycle |
| | 3. Movement and posture and Relaxation | Session 6: Movement and posture<br>Session 7: Breathing and relaxation (focusing the mind) |
| 2<br>Doing something about your life with pain | 4. Dealing with unhelpful, negative thoughts and barriers to change | Session 8: Reflections from day one<br>Session 9: Identifying problems, goal setting and action planning<br>Session 10: Unhelpful thinking and automatic thoughts |
| | Lunch | |
| | Taster activity | Hand massage |
| | 5. Making pain more manageable | Session 11: Barriers to change, challenging unhelpful thoughts. Pros and cons of chronic pain and re framing<br>Session 12: Attention control and distraction<br>Session 13: Identifying things that make pain more manageable |
| | 6. Movement and Relaxation | Session 14: Movement and balance<br>Session 15: Breathing, Relaxation and visualisation |
| 3<br>Communication and relationships | 7. Communication skills | Session 16: Reflections from day 2<br>Session 17: Communication with health professionals<br>Session 18: Communication and listening<br>Session 19: Anger, irritability, frustration and chronic pain |
| | Lunch | |
| | Taster activity | Craft |
| | 9. Movement and Relaxation | Session 20: Movement and stretch<br>Session 21: Relaxation and Guided Imagery |
| 4<br>Follow up | 10. The future | Session 22: Reflections<br>Session 23: Managing setbacks |

Teaching and learning methods include, discussion, brainstorming, sharing narratives and experiences, problem solving, watching educational DVDs and role plays, doing activities and trying new thinking techniques, exercises, mind focus and relaxation.

The courses will be run mainly during the school term within school hours to accommodate those with children (10.00am – 2.45/3.00pm). We will also offer courses over weekends to accommodate those who may find it difficult to take time off work.

4.2 Facilities
Courses will be held in easily accessible venues, i.e. with disabled parking and/ or near public transport. The venues may be in medical or community settings pending accessibility and availability. These will be booked in advance of recruitment; we anticipate giving participants a choice of up to three dates and/or venues.

4.3 Recruiting and training course facilitators
Facilitators will be recruited from a variety of sources:

Health care professionals: press releases will be issued to all the relevant professional magazines seeking people who may be interested in the study and in becoming facilitators. They will be asked to send in curriculum vitae to the study team manager who will interview the health professionals on the telephone. The recruitment criteria are: experience with chronic pain patients, articulate, empathic, an interest in psychological aspects of health care and would be able to run at least two courses. If the applicants match these criteria they will be invited to the two-day training course.

Lay facilitators will be recruited via the community interest companies currently providing expert patient self-management programmes. The criteria for lay facilitators will be interest and experience with facilitating self-management or self-help style groups. They must have, or have had, chronic pain and would be able to run at least two courses. If they fit these criteria they will be invited to the two-day training course with the health care professionals.

We will also use the trained and experienced facilitators from the pilot trial and try to pair these experienced facilitators with newly trained facilitators.

The training course will cover the course content, how to facilitate, dealing with difficult situations and what to do if an adverse event occurs.
Those who attend the training course and are evaluated by the study team as competent will be asked to facilitate future COPERS trial courses. During the course they will be required to demonstrate that they are good listeners, empathic, flexible, able to encourage equal participation, able to encourage laughter, able to managed difficult people, and able to summarise sessions and put the course content into a chronic pain context.

4.4 Quality control and intervention treatment drift
We will pair up experienced facilitators with less experienced or inexperienced facilitators to ensure consistency and promote facilitator confidence.
All courses will either be observed or audio recorded for quality control to check the fidelity of the intervention. A checklist of behaviour change techniques will be used and the evaluator will be required to not only assess structure and content but also whether the required behaviour change techniques were delivered. Audio recordings and observations will be used to provide feedback to facilitators where necessary, so they can modify their performance.

4.5 The control intervention
In the pilot study, the control arm received usual care and a pain leaflet, we realized that we needed to provide a more credible, appealing control arm to encourage

participation in the trial. The control arm in this trial will be relaxation, usual GP care, and the 'The Pain Toolkit' (see appendices). The relaxation will be provided in the form of a relaxation pack; an audio CD with instructions for use and the rationale for the benefits of relaxation. The relaxation audio CD pack will also be given to the intervention arm participants as part of the self-management course.

After randomization participants in the control group will receive the relaxation pack and the Pain Tool Kit and asked to practice the techniques on the CD every day for at least 3 weeks (the same duration as the intervention) and as much as they like thereafter. We will then follow these participants up at twelve weeks, six and twelve months.

## 5. Study Procedures

### 5.1 Informed Consent Procedures

We have two consent stages:
1) Consent to be approached by the study team
2) Consent to be part of the study

1) Consent to be approached by the study team/expression of interest
The GPs or responsible clinician will invite those who appear to be suitable in their clinics to be part of the study, either by invitation letter or face to face. If the person is interested they are asked to complete a 'consent to be approached' form. They can either complete this form by adding their contact details and sending this to the study team in a freepost envelope or interested people can telephone, or email, the study team directly. When potentially interested participants contact the study team directly, they will discuss and explain the trial to the person. If the person is interested a study team member will collect their contact details on the appropriate form indicating that the interest was via the telephone.

2) Consent to be in the trial
Consent will be requested for: being in the trial, having the courses audio recorded, use of anonymised data and permission to check health records at 12 months for extracting data about health care resource use. The consent process will be as follows: the expression of interest, either by mailed form or telephone or email, triggers the mailing of: a COPERS cover letter, the patient information sheet, the trial consent form and the baseline questionnaire. If the patient wishes to be part of the study they return their signed trial consent form and the baseline questionnaire.
The participant is then telephoned to:
Introduce the study team
Check consent is valid and informed (at this point the consent form is countersigned by the study team member and confirmed as valid if appropriate).
Check their questionnaires for completeness.
It is at this point that the participants are formally enrolled in the study.
The participant is then randomised and informed of their allocation.
If allocated to the control group, they are told about the process involved, they will sent a relaxation CD with instructions, the Pain tool-kit and to continue with their usual GP care. They will receive further questionnaires at 12 weeks, 6 and 12 months.
If allocated to the intervention, the participant will be offered the opportunity of participating in a course.

### 5.2 Screening Procedures

The screening process is conducted by the clinicians at the invitation stage of the trial. Those patients identified by electronic searches will be checked by the clinicians responsible for their care to ensure that they are eligible to be in the study. Those patients who respond to the COPERS adverts, who contact the study team directly will be asked questions about their health by the study team to ensure that they are eligible for inclusion.

5.3 Randomisation Procedures

Participants will be randomized once the study team have validated that consent is informed and received and checked the potential participant's baseline questionnaire. Randomisation will be 1.33:1 to the intervention and the control (see sample size calculations for explanation).

All parties will be blind to allocation status up to the point that the allocation is made. Randomisation allocation concealment will be ensured because all data about each potential participant will be collected and checked and once this process is complete the study team will then randomise the patients.

Randomisation will be overseen and implemented by Pragmatic Clinical Trials Unit (PCTU) at Queen Mary University of London via a real time randomisation website. The study team will use the web based randomisation programme, they will confirm eligibility, and input each participants study number, gender and source of recruitment. The PCTU will be responsible for the random allocation website which the study team will log into. Randomisation will be done whilst on the telephone to the participant to avoid having to call the participant again.

5.4 Schedule of Treatment

The intervention will take place over 3 days (10.00 – 14.45/15.00) in one week, with a two-hour follow up session two-weeks later. We will aim to get people on courses within 8 weeks. We are running the delivery of courses in phases over 12 weeks and anticipate that all courses would start within eight weeks from randomisation.

.

5.5 Schedule of Assessment

Baseline questionnaire

↓

Randomisation to Intervention or Control

↓

12 weeks self-efficacy questionnaire

↓

6 month follow-up questionnaire

↓

12 month follow up questionnaire and health resource use data collection via clinical records

5.6 Follow-up Procedures

Participants will receive further follow-up questionnaires at 12 weeks, six and 12 months post randomisation. All the participants will receive a self-efficacy questionnaire at 12 weeks post randomisation, followed by a complete questionnaire at 6 and 12 months. These questionnaires will contain the same measurement tools as the baseline questionnaire. At 12 months (with prior written and signed consent) the study team will also examine the participants' clinical records and extract data

about : co-morbidities, number of consultations with GPs, nurses, hospital admissions, referrals, tests and prescribing data to assess health resource use.
If a participant is unduly delayed in receiving the intervention by more than 12 weeks post randomisation, we will adjust the follow-up interval according to the delay so that the follow-up periods are representative.
We propose to send participants a £5 'high street shop' voucher that is redeemable in multiple stores with their 6 month and 12 month questionnaires to encourage response rates. We propose to give the vouchers on a non-conditional basis. This expression of appreciation has been shown to improve questionnaire return rates. A systematic showed that 'the odds of response were more than doubled when a monetary incentive was used (odds ratio 2.02; 95% confidence interval 1.79 to 2.27) and almost doubled when incentives were not conditional on response (1.71; 1.29 to 2.26).' (Edwards et al BMJ 2002 May 18; 324 (7347): 118).

5.7 End of Study Definition
Once 12 month data collection has been complete, and all clinical records have been checked and the data quality controlled for missing items the study team will inform the relevant approval and funding bodies.

5.8 Data Collection and Follow-up
Baseline data collection
We will collect basic demographic data from the participants i.e. age, gender, ethnicity, we will also ask about language fluency, education background, work status, benefit status and co-morbidities at baseline. We will also ask participants to complete seven standardized validated assessment tools. We developed this basket of measures by carrying out extensive literature reviews on validity and reliability and extent of use in other studies. We convened a focus group consisting of two experts in outcome measures, two GPs, two psychologists and two patients. This group selected the most appropriate measures for the pilot trial which have since been tested and are satisfactory to participants in terms of acceptability, completion and compliance.
The questionnaire is contained in the appendix. The tools we selected are:

- CPG (Chronic Pain Grade) (Von Korff 1992)
- HADS (Hospital Anxiety and Depression Scale) (Zigmond and Snaith 1983)
- EQ-5D (Quality of life) (EuroQol.org)
- PSEQ (Pain Self-Efficacy Questionnaire) (Nicholas 1989, 2007)
- CPAQ (Coping Pain and Acceptance Questionnaire) (McCracken 2004)
- HEIQ (Social integration) (http://www.heiq.org.au/)
- Census global health question (ons.gov.uk/census/2011-census)

In addition to the baseline questionnaire the participants will be asked to complete the self-efficacy questionnaire at 12 weeks post randomisation. We are checking self-efficacy at this point to be able to test the hypothesis that change in self-efficacy is a mediator for change in our primary outcomes. We have established in a systematic review that self-efficacy is the best candidate for being an important mediator of treatment effect for self-management interventions. Full questionnaires that include all assessment measures will be posted to participants at 6 months and 12 months post randomisation. There will be one postal reminder and non-responders will be contacted by phone to obtain a minimum dataset, if possible, for primary clinical outcomes only.

Additionally, at the trial consent stage, we will request access to clinical notes at 12 months for data about: number of consultations, referrals, hospital admissions,

prescribing and co-morbidities in the last 12 months. We will measure co-morbidity using each participant's Cumulative Illness Rating Scale (CIRS) score (Huddon et al, 2005). This is a measure of the burden of co-morbidity for each participant.

## 6. Adverse event vigilance

All adverse events will be reported to the study team manager and PIs via the facilitators who deliver the courses to the trial participants either immediately by telephone or by email, pending seriousness.

### 6.1 General Definitions

#### 6.1.1 Adverse Event (AE)

An adverse event in this study would be any untoward physiological or psychological occurrence in a subject to whom the intervention has been administered, including occurrences which are not necessarily caused by or related to the self-management course. An AE can therefore be any unfavourable and unintended sign, symptom or disease temporarily associated with study activities. In this study an example would be a course participant becoming unduly upset during the course and having to leave the course temporarily to compose themselves. Experience form the pilots suggests that such AEs are very rare.

#### 6.1.2 Serious Adverse Event (SAE)

A serious adverse event in this study would be:

- Death
- Life threatening
- Requires hospitalisation
- Results in persistent or significant disability or incapacity
- Is otherwise considered medically significant by the investigator.

Due to the nature of the intervention being evaluated in this trial (a non pharmacological, group based self management course) we believe that a SAE is extremely unlikely in this study. If a course participant experiences a SAE, the facilitators will notify the study manager and chief investigators immediately, the study team would then inform the Research Ethics Committee, if in the opinion of the chief investigator the event was:

a) Related to the COPERS intervention.
b) Unexpected and possibly related to the COPERS intervention.

The Principal Investigator determines whether the adverse event is serious enough to refer on, cases that are unexpected and possible related need to be submitted within 15 days of the chief investigator becoming aware of them. In any case all details must be reported and recorded and stored in the trial master file.

#### 6.1.3 Other adverse events

Other incidents/events that are not defined as serious will be reported by the facilitators or the study team to the study manager and recorded in the Trial Master File and followed up by the research team. All adverse events will be documented in the participants' case report form (CRF).

Non-serious adverse events may include incidences that are:

a) Related to COPERS
b) Unexpected but unrelated to COPERS

(http://www.nres.npsa.nhs.uk/applications/after-ethical-review/safetyreports/safety-reports-for-all-other-research/)

6.2 Notification and reporting Adverse Events or Reactions

Serious Adverse Event (SAEs) that are considered to be 'related' and 'unexpected' will be reported to the sponsor within 24 hours of learning of the event and to the Main REC within 15 days in line with the required timeframe. Other adverse events will be documented in the Trial master File and followed-up by the study team to ensure risk is minimised in the future and that the welfare of participants are monitored.

6.3 Annual Safety Reporting

The Chief Investigator will send an Annual Progress Report to the main REC and to the sponsor and include information about adverse events reported.

6.4 Overview of the Safety Reporting Process/ responsibilities

The adverse event protocol reporting and responsibility procedures are contained in the appendices.

The Chief Investigator will ensure that adverse event vigilance monitoring and reporting is conducted in accordance with the sponsor's requirements.

6.5 General Safety Issues

This is a study with relatively low risk, it is non-invasive and non-pharmacological. The techniques that we teach the participants can be used, or not, by the participants, we regard all of the techniques taught as low risk in terms of either psychological or physical harm. The main concern is with participants who endeavour to do too much potentially putting themselves at risk of more pain, however we build in SMART (simple, measurable, achievable, realistic and timed) goals to counter this. Other than this we encourage heightened awareness of negative thoughts and propose ways of dealing with these. The long term repercussions of this are highly unlikely to be negative. We are not aware of any serious adverse events occurring in studies of this nature in either the short or long term. However the study team are available at any point during the study duration to advise participants should they experience any difficulties as a result of being in the trial. Additionally participants can contact the Patient Advice and Liaison Services (PALS) or the Complaints Officer at the University, should they deem it necessary (all this information is contained in the Patient Information Leaflet)

## 7. Statistical Considerations

7.1 Primary outcomes

The primary effectiveness outcome for this study is pain related disability. These data will be collected by postal and email survey at six months and 12 months. Twelve months is the primary endpoint for this analysis.

The primary health economic outcome is the Incremental Cost Utility Ratio (ICUR). Quality of life using the EQ5d data will be collected at baseline, six and 12 months. Health resource use data will be collected from patient records at 12 months. Twelve months is the primary endpoint for this analysis.

When the final data have been input and cleaned and checked, the data base will be locked and no further entries and or amendments permitted.
Descriptive statistics will be produced to detail the baseline characteristics of each treatment arm.
For Pain related disability we will calculate the mean change scores (and standard deviation) for each arm of the trial from 0-6 months and 0-12 months.

## 7.2 Secondary outcomes

Secondary outcomes will be input at the same time as the primary outcomes. The database will be locked down when all data have been input, checked and cleaned. We will use a 5% level of statistical significance and assess change scores at 0-6 and 0-12 months.
We will collect additional self-efficacy data at 12 weeks. Our rationale for conducting this additional data collection is that self-efficacy is a predictor of effectiveness but change in self-efficacy is a mediator. If we find no differences in our primary outcome we will carry out a mediator analysis to see if those who did change in self-efficacy did significantly better. As we have developed our intervention from an evidence and theory base, our proof of principle would be change in self-efficacy in the treatment group post intervention but not in the control group.

## 7.3 Sample Size

We estimate that the self-management intervention group intra-cluster correlation coefficient will be in the region of 0.1. Our pilot study showed that a realistic and optimal course group size is approximately 12 (range between 10 and 15). However, we expect a loss to follow-up of 25%, giving a mean endpoint group size of 9 in the intervention arm.
We assume that the total variance in the intervention arm is 10% greater than in the control arm and that we require a power of 80% at the 5% significance level to detect a medium-small effect size of 0.3 (Moerbeck and Wong 2008) in our pain related disability measure. This is commensurate with the largest change seen in a recent systematic review on EPP (Foster 2007), and also with the sort of change effected by interventions for chronic back pain on any continuous outcome measure. According to the method of Moerbeek and Wong (2008), the optimum design will require 288 patients in the intervention arm and 218 in the control arm. Assuming a non-differential loss to follow-up of 25% (in both trial arms), the total sample size will need to be inflated from 506 to 673 patients. The requirement will be 32 therapy groups of 12 patients and 289 individual controls i.e. 1.33 intervention patients for every control.
This 1.33:1 ratio (intervention/control) may also help improve recruitment to the study if patients are told that they are more likely to receive the active treatment.

We will recruit in four phases over 14 months.

We aim to send invitations in May 2011, September 2011, January 2012, March 2012 (and complete courses by July 2012)

Phase 1 May – July 2011: 2 courses in Warwick and 2 in London = 4
Phase 2 Sept -Dec 2011: 5 courses in Warwick and 5 in London = 10
Phase 3 Jan – March 2012: 5 courses in Warwick and 5 in London = 10
Phase 4 April – July 2012: 4 courses in Warwick and 4 in London = 8

Total 32 courses, however we will allow for 36 courses and run four additional courses in each centre in the final phase, if necessary.

Previous research and our pilot study indicated that around 5% of the adult population have chronic musculoskeletal pain. Of these 10% may be interested in participating in the trial and around half of these will be recruited to the study i.e. 0.25% of adults, or 0.175% of total population (assumes 70% are adults, in Tower Hamlets 78% of the population are adults (October 2010). This means that to recruit our 673 participants we need a population base of around 399,000, or around 55 practices with an average total list size of 7,000. This may be an overestimate of the number of practices needed as it does not account for participants recruited from pain services and advertisements within general practices. We will refine these recruitment estimates during the initial wave of recruitment to arrive at final number of practices required

### 7.4 Statistical Analysis

A full analysis plan will be drawn up and agreed by the trial statistician and chief investigators prior to any data analysis.

Descriptive statistics will be used to summarise the characteristics of participants in each arm of the trial.

We will analyse change scores from baseline and follow up data in both arms and compare the level of change between the two groups using a linear mixed-effects model that accommodates both the clustering of patients within therapists in the intervention arm and the individually randomised patients in the control arm.

Where participants withdraw we will compare the characteristics of those withdrawing against those who remain in the study.

Where data are missing we will carry out multiple imputation to enable us to conduct an intention-to-treat analysis. We will also conduct an available case analysis and a complier-average causal effect (CACE) analysis.

To estimate treatment effects we envisage using a mixed effects model with fixed effects for intervention group, age, gender, centre (London or Warwick) and baseline value of outcome, and random effect for course. We will use STATA to analyse the data. We will report the standardised mean differences, numbers needed to treat (NNTs) and relative risks for a 30% change/improvement for our primary and secondary outcomes (Ostello et al, 2008).

## 8. Data Handling & Record Keeping

8.1 Confidentiality

Information with regards to study patients will be kept confidential and managed in accordance with the Data Protection Act, The Research Governance Framework for Health and Social Care and Research Ethics Committee Approval.

We will hold paper and electronic data about the participants, to include:
name, address, date of birth and contact telephone numbers.

Additionally we will collate demographic data and questionnaire data about quality of life, anxiety and depression, self confidence, coping, social integration and pain.

We will hold paper copies of all participant contact details at Barts and the London school of Medicine and Dentistry; these will be kept in locked filing cabinets in a secure office. Data about participants recruited in Warwick will be held locked filing cabinets at Warwick Medical School.

All paper copies of participant questionnaires will be kept in locked filing cabinets at Barts and the London school of Medicine and Dentistry only.

At 12 months we will hold data about health resource use and co-morbidities as well.

All participant information gathered from the questionnaires will be pseudo-anonymised and only identified by a participant ID number.

The master coding for participant ID number will be held on an encrypted and password protected memory stick and in hard copy form in a secure locked filing cabinet, in a security controlled environment in a locked study team room. Only the immediate study team members will have access to this information.

All electronic data will be entered into databases with complete audit trails by the Pragmatic Clinical Trials Unit at Queen Mary University of London. All computerised participant identifiable information is held on a non-web linked separate computer, which is password protected, only key members of the study team will have access to this: the PI and the COPERS study team researchers who will input and audit the data. Thus all follow-up data will be managed in London. All information will be backed up and on an encrypted and password protected memory stick that is stored in a secure off site location.

- The Chief Investigators will be the ‘Custodians’ of the data.
- All patients will be anonymised with regards to any future publications relating to this study.

8.2 Study Documents

These essential study documents will be organized, maintained and filed in the Trial Master File.

- A signed protocol and any subsequent amendments
- Intervention detail (the course facilitator manual)
- Sponsor Self-Monitoring template for the trial team to complete on a regular basis as detailed by the Monitoring section
- Patient Information Sheets
- Consent Forms
- Indemnity documentation from sponsor
- Conditions of Sponsorship from sponsor
- Conditional/Final R&D Approval
- Signed site agreement
- Ethics submissions/approvals/correspondence
- CVs of CI and site staff
- Delegation log
- Staff training log

- Site signature log
- Patient identification log
- Screening log
- Enrolment log
- Monitoring visit log
- Protocol training log
- Correspondence relating to the trial
- Communication Plan between the CI/PI and members of the study team
- SAE reporting plan for the study

8.3 Case Report Form (CRF)

The CRF for this study will be maintained and produced by the study administrators at Warwick and London. The CRF will record: name, address, date of birth, GP details, participant number, registration of interest date, eligibility/exclusion criteria checklist, randomisation date and outcome, consent date, questionnaire receipt dates, course details and dates, dates control information sent, any study intervention delays, withdrawal from study, follow up of outcomes, death, special needs or requirements and adverse events. These data will be kept on an encrypted file in a stand-alone PC, for security purposes.

8.4 Record Retention and Archiving

During the course of research, all records will be kept in secure conditions. When the research trial is complete, the records will be archived for a further 20 years by BLT or QMUL, in their approved repository for long-term storage of local records (the Trust Modern Records Centre which is based at 9 Prescot Street).

8.5 Compliance

The Chief Investigator will ensure that the trial is conducted in compliance with the principles of the Declaration of Helsinki (1996), and in accordance with all applicable regulatory requirements including but not limited to the Research Governance Framework, Trust and Research Office policies and procedures and any subsequent amendments.

8.6 Clinical Governance Issues

8.6.1 Ethical Considerations

This protocol and any subsequent amendments, along with any accompanying material provided to the patient, in addition to any advertising material will be submitted by the Investigator to an Independent Research Ethics Committee. Once written approval from the Committee has been granted it will be subsequently submitted to the JRO to obtain Final R&D approval.

Ethical considerations pertaining to recruitment are minimal but centre around access to patient information. Only the clinic staff, PCRN and research staff (subject to the appropriate permissions) will be able to search GP registers and invite suitable patients to participate. Initial contact will be via the patient's clinician only. Patients will have the choice whether or not to participate. They will receive one reminder letter and subject to resources and or approvals they may be followed up with a phone call.
The risks to the participants in this study are low, however the study team are aware that the course can trigger emotional reactions. We have therefore ensured that our facilitator training course trains facilitators in distress management. Each course has two facilitators so should any participant become unduly distressed they can be helped by a facilitator who will, if necessary, and with the participants agreement

withdraw the participant from the group and help them until they are ready to return to the group, go home or seek further help from a more suitable health care professional. Under no circumstances will a participant be left alone whilst distressed. If the facilitator feels that the participant is a danger to themselves or others, they will seek permission to contact the participant's GP or take them to A&E.

We will also ensure that a member of the study team is always available by mobile telephone for the duration of any course should any emergency advice be needed. The study team will have a list of clinically qualified personnel to call on should it be necessary. Both chief investigators are General Practitioners and we will recruit clinical volunteers to be on call whilst the courses are running.

8.7 Quality Control and Quality Assurance

8.7.1 Summary Monitoring Plan
Study reports will be produced for the funder, the NIHR, BLT/QMUL Joint Research Office and Ethics. Additional Audits and inspections will be carried out by the Pragmatic Clinical trials Units (QMUL), the funder and BLT/QMUL Joint Research Office if necessary.

8.7.2 Audit and Inspection
A systematic and independent examination of trial related activities and documents will be conducted by the Pragmatic Clinical Trials Unit at Queen Mary University of London after three months and then pending a risk assessment at 12 monthly intervals. These audits will determine whether the trial related activities are conducted, and the data recorded, analysed and accurately reported according to the protocol, sponsor's standard operating procedures (SOPs), Good Clinical Practice (GCP), and the applicable regulatory requirement(s)

8.8 Non-Compliance
In the event of non-compliance with regulations, the sponsor (BLT/QMUL JRO) will maintain a log of the non-compliances to ascertain if there are any trends developing which may escalate. The sponsor will assess the non-compliances and action a timeframe in which they need to be dealt with. Each action will be given a different timeframe dependant on the severity. If the actions are not dealt with accordingly, the JRO will agree an appropriate action, including an on-site audit.

## 9. Trial Committees
The Trial Management Group (TMG) is responsible for overseeing the day to day management of the trial. The Trial Steering Committee (TSC) is responsible for checking protocol adherence, ratifying protocol amendments and monitoring the integrity of the trial. They are also responsible for considering the recommendations made by the Data Monitoring and Ethics Committee (DMEC). A DMEC will also be convened. A summary of the TMG, TSC and DMEC responsibilities and members to date are detailed in the appendix.
The DMEC will have ultimate responsibility for recommending early termination of the trial. The DMEC recommendations will be considered by the TSC and ultimately carried out by the TMG. Early termination of the trial will be considered if there is either clear benefit or harm of the intervention or futility of the treatment. Trial data and new external evidence of harm or futility of continuing from other sources will be used to inform the decision.

## 10. Publication Policy

The data from the study will be used to write a detailed monograph for the funder and papers to be published in peer reviewed journals. Results will also be disseminated via conferences.

## 11. References


Carnes D, Homer K, Miles C, Pincus T, Rahman A, Underwood M, Taylor S. Effectives components and characteristics of self-management courses for chronic pain. (Submitted and under review for Eur J Pain)

Croft P. Is life becoming more of a pain? BMJ 2000;320:1552-53.

Department of Health. 'Improving Chronic Disease Management' – a DH policy paper, March 2004

Expert Patient Programme. www.expertpatients.co.uk/public/default.aspx (accessed 25.02.08)

Foster G, Taylor SJC, Eldridge SE, Ramsay J, Griffiths CJ. Self-management education programmes by lay leaders for people with chronic conditions. Cochrane Database of Systematic Reviews 2007, Issue 4. Art. No.: CD005108. DOI:10.1002/14651858.CD005108.pub2.

Huddon C, Fortin M, Vanasse A. Cumulative Illness Rating Scale was a reliable and valid index in a family practice context. J Clin Epidemiol. 2005; 58:603-8.

International association for the study of pain. Pain 1986;Suppl 3:s1-s226.

Kennedy A, Reeves D, Bower P, Lee V, Middleton E, Richardson G, et al. The effectiveness and cost effectiveness of a national lay-led self care support programme for patients with long-term conditions: a pragmatic randomised controlled trial. J Epid Comm Health 2007;61:254-61.

Maniakadis N, Gray A. The economic burden of back pain in the UK. Pain 2000; 84:95-103.

McCracken LM, Vowles KE, Eccleston C. Acceptance of Chronic Pain: component analysis and a revised assessment method. Pain 2004; 107: 159-66

Moerbeek, M. & Wong, W.K. Sample size formulae for trials comparing group and individual treatments in a multilevel model *Statist. Med.* 2008;27:2850-2864

Nicholas MK. The pain self-efficacy questionnaire: taking pain into account. Eur J Pain 2007; 11:153-63

Ostello RW, Deyo RA, Stratford P, et al. Interpreting change scores for pain and functional status in low back pain: towards an international consensus regarding minimally important change. Spine 2008; 33: 90-94

Parsons S, Breen A, Foster NE, et al. Prevalence and comparative troublesomeness by age of pain in different body locations amongst those reporting chronic pain. Fam Pract 2007;24: 308-16.

Prime minister speech 7 January 2008 Speech on National Health Service. http://www.pm.gov.uk/output/Page14171.asp, (accessed 24/02/08)

Secretary of state for Health. The expert patient: a new approach for chronic disease in the 21st century. London: Stationery Office, 2001. www.dh.gov.uk/en/publicationsandstatistics/publications/publicationspolicyandguidance/DH_4006801

Von Korff M, Ormel J, Keefe F, Dworkin S. Grading the severity of chronic pain. Pain 1992; 50: 133-149

Wanless D. Securing Our Future Health: Taking a Long-Term View. Final Report. HM Treasury, April 2002. www.hm-treasury.gov.uk/Consultations_and_Legislation/wanless/consult_wanless_final.cfm , accessed 01/ 01/ 2008.

Zigmond AS, Snaith RP. The hospital anxiety and depression scale. Acta Psychiatr Scand 1983; **67(6):** 361–370.

## 12. Appendices

I) Trial Organisation chart
II) GP/Clinic patient invitation letter with consent to approach form
III) GP/Clinic patient invitation reminder letter
IV) Patient Information Leaflet
V) Trial Consent Form
VI) Advertisements
VII) Questionnaires
VIII) Trial serious adverse event reporting
IX) TMG, TSC and DMEC summary terms of reference

(For appendices II) to VII) see attachments)

Appendix I – Trial Organisation Chart

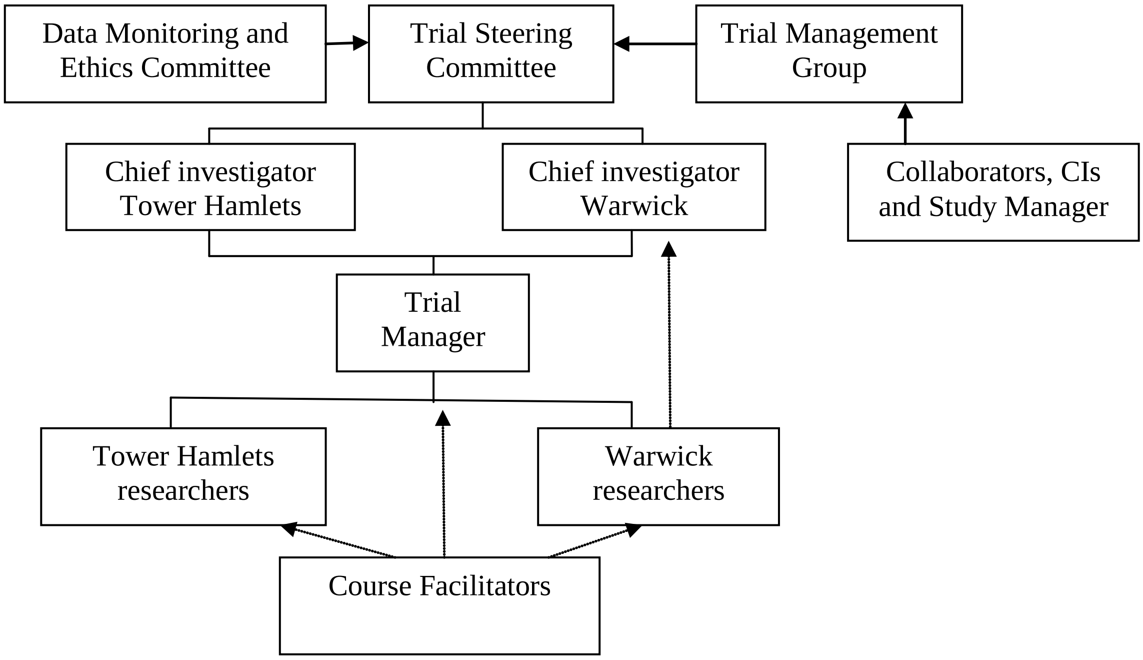

Appendix VIII – Information with regards to Safety Reporting in Non-CTIMP Research

| | Who | When | How | To Whom |
|---|---|---|---|---|
| **SAE** | Chief Investigator | -Report to Sponsor within 24 hours of learning of the event<br><br>-Report to the MREC within 15 days of learning of the event | SAE Report form. | Sponsor and MREC |
| **Urgent Safety Measures** | Chief Investigator | Contact the Sponsor and MREC Immediately<br><br>Within 3 days | By phone<br><br>Substantial amendment form giving notice in writing setting out the reasons for the urgent safety measures and the plan for future action. | Main REC and Sponsor<br><br>Main REC with a copy also sent to the sponsor. The MREC will acknowledge this within 30 days of receipt. |
| **Progress Reports** | Chief Investigator | Annually ( starting 12 months after the date of favourable opinion) | Annual Progress Report Form | Main REC |
| **Declaration of the conclusion or early termination of the study** | Chief Investigator | Within 90 days (conclusion)<br><br>Within 15 days (early termination) | End of Study Declaration form | Main REC with a copy to be sent to the sponsor |
| **Summary of final Report** | Chief Investigator | Within one year of conclusion of the Research | Include whether the study has met its objectives, main findings dissemination of results | Main REC with a copy to be sent to the sponsor |

**Appendix IX**

**COPERS RCT, Trial Oversight Committees: Summary of Terms of Reference**
(Detailed terms of references to be implemented are produced and located at the Pragmatic Clinical Trials Unit at Queen Mary University of London, http://www.ihse.qmul.ac.uk/chs/Docs/25919.pdf)

**Trial Management Group**

**Purpose:** The Trial management Group (TMG) is responsible for overseeing the day to day management of the trial, to monitor and manage all aspects of the conduct and progress of the trial.

The TMG will consider and act on the recommendations of the Trial Steering Committee (TSC) and Data Monitoring and Ethics Committee (DMEC).
The TMG will include both CIs (Martin Underwood and Stephanie Taylor as Chair), trial statistician (Stephen Bremner), study manager/coordinator (Dawn Carnes), data manager (Sandy Smith) and collaborators.

**Responsibilities**:

- Ensuring the protocol is adhered to and take action, as necessary, to remedy any difficulties
- Consider and act on the recommendations of the TSC and DMEC
- Refer any possible protocol amendments to the TSC
- The TMG will meet frequently monthly or as required by the progress of the trial and determined by the members of the group.

**Trial Steering Committee**

**Purpose:** The role of the Trial Steering Committee will be to provide overall supervision of the trial on behalf of the Trial Sponsor and Trial Funder to ensure trial is conducted in accordance with the Principles of Good Practices relevant regulations, monitor the progress of the trial, adherence to protocol and patient safety.

The TSC will include an independent chair, a pain specialist and a trialist, both Chief Investigators, two lay representatives and an independent statistician.

**Responsibilities:**

- Consideration of new information of relevance from other sources
- Making executive decisions about the trial e.g. protocol amendments as suggested by the TMG
- Consider and act on the recommendations of the DMEC

**Data Monitoring and Ethics Committee**

**Purpose:** The role of the DMC will be to review the accruing trial data and to assess whether there are any ethical or safety issues why the trial should not continue. The DMEC will also be asked by the TSC, Trial Sponsor or Trial Funder to consider data emerging from other studies. The DMEC should report to the TSC.

The DMEC will be independent of the investigators and the funder/sponsor.
It will consist of a chair, one clinician experienced in the clinical area**,** one expert trial statistician.

**Responsibilities:**

- Formalising appropriate procedures for reporting of adverse events
- Determining a schedule of meetings at least annually and timed so that reports can feed into TSC meetings
- Make recommendations to the TSC and/or TMG.

(If an unblinded interim statistical analysis is required this will be carried out by a qualified statistician independent of the trial)
