# Supplementary material for: Novel Three-Day, Community-Based, Nonpharmacological Group Intervention for Chronic Musculoskeletal Pain (COPERS): A Randomised Clinical Trial
Source: PLoS Med. 2016 Jun 14;13(6):e1002040. doi: 10.1371/journal.pmed.1002040 (PMC4907437; doi:10.1371/journal.pmed.1002040)
Supplement: S1 Approval — (PDF) [file pmed.1002040.s003.pdf]

NHS
National Research Ethics Service
Cambridgeshire 4 Research Ethics Committee
Victoria House
Capital Park
Fulbourn
Cambridge
CB21 5XB

Telephone: 01223 597653
Facsimile: 01223 597645

18 March 2011

Prof Stephanie Taylor
Professor of Public Health and Primary Care
Queen Mary University of London
Centre for Health Sciences
2 Newark St
London
E1 2AT

Dear Prof Taylor

**Study Title:**	**COping with persistent Pain, Effectiveness Research for Self-management: a randomised controlled trial**
**REC reference number:**	**11/EE/0046**

Thank you for your letter of 14 March 2011, responding to the Committee's request for further information on the above research and submitting revised documentation.

The further information has been considered on behalf of the Committee by the Chair who felt that this was an extremely thorough and comprehensive response to the post-review letter, for which the researchers should be commended.

Confirmation of ethical opinion

On behalf of the Committee, I am pleased to confirm a favourable ethical opinion for the above research on the basis described in the application form, protocol and supporting documentation as revised, subject to the conditions specified below.

Ethical review of research sites

The favourable opinion applies to all NHS sites taking part in the study, subject to management permission being obtained from the NHS/HSC R&D office prior to the start of the study (see "Conditions of the favourable opinion" below).

The Committee has not yet been notified of the outcome of any site-specific assessment (SSA) for the non-NHS research site(s) taking part in this study. The favourable opinion does not therefore apply to any non-NHS site at present. I will write to you again as soon as one Research Ethics Committee has notified the outcome of a SSA. In the meantime no study procedures should be initiated at non-NHS sites.

Conditions of the favourable opinion

The favourable opinion is subject to the following conditions being met prior to the start of the study.

Management permission or approval must be obtained from each host organisation prior to the start of the study at the site concerned.

For NHS research sites only, management permission for research ("R&D approval") should be obtained from the relevant care organisation(s) in accordance with NHS research governance arrangements. Guidance on applying for NHS permission for research is available in the Integrated Research Application System or at http://www.rdforum.nhs.uk.

Where the only involvement of the NHS organisation is as a Participant Identification Centre (PIC), management permission for research is not required but the R&D office should be notified of the study and agree to the organisation's involvement. Guidance on procedures for PICs is available in IRAS. Further advice should be sought from the R&D office where necessary.

Sponsors are not required to notify the Committee of approvals from host organisations.

It is the responsibility of the sponsor to ensure that all the conditions are complied with before the start of the study or its initiation at a particular site (as applicable).

Approved documents

The final list of documents reviewed and approved by the Committee is as follows:

Document	*Version*	*Date*
Protocol	Version 10	10 January 2011
Letter of invitation to participant: from GP/Clinician	Version 2	14 March 2011
Letter of invitation to participant: GP invitation letter	Version 1	14 March 2011
GP/Consultant Information Sheets	Version 3	14 March 2011
Response to Request for Further Information: from Dawn Carnes		14 March 2011
Investigator CV: Dr Stephanie Taylor		24 January 2011
Questionnaire: Follow Up Questionnaire at 8 weeks	Version 5	14 March 2011
Questionnaire: Follow Up Questionnaire at 6 months	Version 5	14 March 2011
Questionnaire: Follow Up Questionnaire at 12 months	Version 5	14 March 2011
Trial Flow Chart	Version 1	03 February 2011
Participant invitation reminder letter	Version 3	14 March 2011
Participant Consent Form: - consent to approach	Version 1	29 November 2010
Participant Information Sheet	Version 4	14 March 2011
Questionnaire	Version 2	24 January 2011
Questionnaire: Baseline	Version 5	14 March 2011
Advertisement: "Are you an adult with long term pain in your muscles and joints?"	Version 1	29 November 2010
Referees or other scientific critique report: Peer Review, Professor Robert Walton		18 January 2011
Evidence of insurance or indemnity: Newline (Policy No:Q31000193) (01.08.10 to 01.08.11)		04 August 2010
REC application: Submission code: 69838/185916/1/162		02 February 2011
Participant Consent Form: consent to be in trial	Version 4	14 March 2011
Covering Letter: from Dawn Carnes		04 February 2011
Letter from Sponsor : Barts and The London		03 February 2011
Letter from Funder - NIHR		20 October 2008

Statement of compliance

The Committee is constituted in accordance with the Governance Arrangements for Research Ethics Committees (July 2001) and complies fully with the Standard Operating Procedures for Research Ethics Committees in the UK.

After ethical review

Now that you have completed the application process please visit the National Research Ethics Service website > After Review

You are invited to give your view of the service that you have received from the National Research Ethics Service and the application procedure. If you wish to make your views known please use the feedback form available on the website.

The attached document *"After ethical review – guidance for researchers"* gives detailed guidance on reporting requirements for studies with a favourable opinion, including:

- Notifying substantial amendments
- Adding new sites and investigators
- Progress and safety reports
- Notifying the end of the study

The NRES website also provides guidance on these topics, which is updated in the light of changes in reporting requirements or procedures.

We would also like to inform you that we consult regularly with stakeholders to improve our service. If you would like to join our Reference Group please email referencegroup@nres.npsa.nhs.uk.

**11/EE/0046**	**Please quote this number on all correspondence**

With the Committee's best wishes for the success of this project

Yours sincerely

Dr Leslie Gelling
Chair

Email: susan.davies@eoe.nhs.uk

Enclosures: "After ethical review – guidance for researchers" [*SL- AR2]*
